# Spatial Distribution Pattern of *Aromia bungii* Within China and Its Potential Distribution Under Climate Change and Human Activity

**DOI:** 10.1002/ece3.70520

**Published:** 2024-11-13

**Authors:** Liang Zhang, Ping Wang, Guanglin Xie, Wenkai Wang

**Affiliations:** ^1^ Institute of Entomology, College of Agriculture Yangtze University Jingzhou China; ^2^ MARA Key Laboratory of Sustainable Crop Production in the Middle Reaches of the Yangtze River (Co‐Construction by Ministry and Province), College of Agriculture Yangtze University Jingzhou China

**Keywords:** climate change, human activity, MaxEnt model, MigClim model, spatial autocorrelation

## Abstract

*Aromia bungii* is a pest that interferes with the health of forests and hinders the development of the fruit tree industry, and its spread is influenced by changes in abiotic factors and human activities. Therefore, exploring their spatial distribution patterns and potential distribution areas under such conditions is crucial for maintaining forest ecosystem security. This study analyzed the spatial differentiation characteristics of the geographic distribution pattern of *A. bungii* in China using Moran's *I* and the Getis‐Ord General *G* index. Hot spot distribution areas were identified using Getis‐Ord Gi*. An optimized MaxEnt model was used to predict the potential distribution areas of *A. bungii* within China under four shared economic pathways by combining multivariate environmental data: (1) prediction of natural environmental variables predicted under current climate models; (2) prediction of natural environmental variables + human activities under current climate models; and (3) prediction of natural environmental variables under the future climate models (2050s and 2070s). Meanwhile, MigClim was used to simulate the unoccupied suitable area in the presence of obstacles under future climate change. The results showed that human activities, minimum temperature of the coldest month, and precipitation of the wettest month had positive effects on the distribution of *A. bungii*. However, in the current period, human activities drastically reduced the survival area of *A. bungii*, and its suitable distribution area was mainly concentrated in the eastern and central regions of China. Under the influence of climate change in the future, the habitat of *A. bungii* will gradually increase. Additionally, the MigClim model indicates that the area unoccupied by *A. bungii* has been on a continuous increasing trend. This study provides a positive reference for the prevention and control of *A. bungii* and the maintenance of forest health and ecosystem security, and provides important theoretical guidance for researchers, policymakers, and governments.

## Graphical abstract

This study employs spatial geography and species distribution modeling to investigate potential trends in *Aromia bungii* under future climate conditions. The findings contribute to a deeper comprehension of the distribution of *A. bungii* and furnish invaluable insights for the maintenance of forest health and the security of ecosystems. Furthermore, the findings have significant implications for researchers, policymakers, and governments.

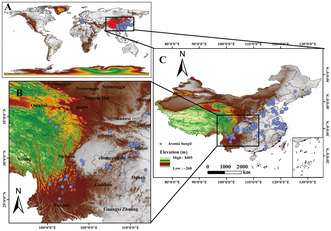

## 1 Introduction

Climate change and human activities have significant implications for global biodiversity and ecosystem sustainability (Zhao et al. 2023). With the intensification of the greenhouse effect, global average surface temperatures are projected to reach or even exceed 1.5°C in the coming period (within 20 years). At the same time, climate change is intensifying the hydrological cycle, which is bringing more intense precipitation and flooding, however, there are also many areas that are also facing more severe droughts (Raffa et al. 2023; Schleussner, Trisos, and Mukherji 2023). Climate change can accelerate impacts on species range distribution patterns, including species extinctions, changing predator–prey dynamics, reductions in biodiversity, loss of ecosystem resilience, and unobserved associated impacts (Lu et al. 2024; Wang et al. 2024). Meanwhile, as human societies develop, the extent, mode, and intensity of human activities can lead to changes in land‐use types, which in turn will generate different types of new landscape interfaces that can directly (or indirectly) affect the spatial distribution and diversity of species (Gallardo et al. 2017; Maiorano et al. 2019). Human activities have not only led to habitat fragmentation and loss, but have also further compressed the environment suitable for species through development, urban sprawl, and agricultural expansion. In addition, frequent resource use reduces the adaptive and dispersal capacity of species, yet transport and trade in the context of globalization provide new routes for the long‐distance spread of certain species, increasing the potential for their transregional dispersal (Marshall, Banks, and Cook 2014; Hemmingmoore et al. 2020). To address these challenges, research on the impacts of climate change and human activities on the geographical distribution patterns of species can help to develop scientifically effective conservation measures.

As an important part of the agroecosystem, fruit trees not only provide abundant food resources for human beings, but also play an important role in maintaining ecological balance, protecting biodiversity, and regulating climate (Huang et al. 2020; Zhao et al. 2024). However, due to climate change and excessive interference from human activities, the pests have caused continuous harm to China's fruit tree ecosystems, bringing great losses to the ecological security of fruit trees as well as to the socioeconomics of society (Kärvemo et al. 2014). *Aromia bungii* (Faldermann, 1835), belonging to the subfamily Cerambycinae of the family Cerambycidae, which is an important stem‐boring pest of fruit trees and ornamental trees of the Rosaceae family, causing damage to over 50 species of trees (Men et al. 2019; Chen et al. 2024). *Aromia bungii* is native to East Asia and has a wide distribution in countries such as North Korea, South Korea, Vietnam, and Russia. In China, *A. bungii* is distributed throughout the rest of the country, except Xinjiang and Xizang, and particularly harms fruit tree species, including apricots, cherries, peaches, and plums, in the central, eastern, and southern China, with particular attrition rates of up to 90% on peach trees (Zou et al. 2019). The eggs of *A. bungii* are typically laid in the epidermal crevices of the trunk, and the larvae feed on the phloem and xylem, forming irregular apertures. This can cause the trunk to become hollow, leading to tree weakness and even death in severe cases (Cao et al. 2022). Due to the long larval cycle, strong concealment, and strong flying ability of the adult, it is not easy to find out in the short term after infestation (Wu et al. 2022; Yamamoto, Ishikawa, and Uehara 2022). In addition, adult males of *A. bungii* lived 47.5 to 48.8 days and females 53.3 to 54.3 days, showing relatively long lifespans, a characteristic that has important implications for their dispersal and damage patterns. *Aromia bungii* destroys the health of forests, seriously harms the sustainable development of ecosystems, and causes great economic losses and ecological disasters to the forest and fruit industries as well as landscaping (Germinara et al. 2019; Sunamura et al. 2021). Therefore, predicting the potential distribution areas of *A. bungii* can provide organizations with scientifically accurate information on migration, which can help to develop preventive measures in a timely manner. Meanwhile, preventing the spread routes of *A. bungii* is crucial to the sustainable development of orchard ecological security and human health.

Species distribution models (SDMs), also known as ecological niche models (ENMs), are mathematical models that estimate the probability of a species occurring in a suitable area based on actual distribution data and spatial environmental variables (Gallien et al. 2012; Boiffin, Badeau, and Bréda 2017). With the continuous development of computers and geographic information systems (GIS), SDMs have been continuously improved and refined. SDMs have become widely used for predicting the reconstruction of suitable areas for animals, plants, and microorganisms, as well as their potential distribution areas (Kandel et al. 2015). The MaxEnt model is a commonly used tool for assessing the potential distribution of species (Wang et al. 2020; Low et al. 2021). The algorithm is acknowledged for its ability to make reliable predictions for species with restricted distribution ranges or small sample sizes, giving it an advantage over other algorithms (Remya, Ramachandran, and Jayakumar 2015; Li, Fan, and He 2020).

Previous studies on predicting species distributions have often focused on environmental variables as the main factors influencing changes (Singer et al. 2016; Melo‐Merino, Reyes‐Bonilla, and Lira‐Noriega 2020). However, in addition to environmental variables, it is important to consider that geographic barriers can also limit species dispersal and may result in the real ecological niche of a species being smaller than the theoretical ecological niche (Case et al. 2005; Algar et al. 2013). Species migration modeling can shape species ranges in current and future periods, providing guidance for species to cope with climate change. Additionally, it is critical for understanding population dynamics, predicting the spread of invasive species, and evaluating the conservation status of rare species (Veran et al. 2015; Li et al. 2017). The MigClim model provides a reliable simulation of species dispersal in stable or undisturbed landscapes (Engler, Hordijk, and Guisan 2012; Wang et al. 2019). The model is very flexible as the parameter values can be adjusted to suit the specific situation. Meanwhile, it is compatible with existing SDMs (Alarcón and Cavieres 2018; Benhadi‐Marín, Fereres, and Pereira 2022; Lemes et al. 2022).

Therefore, first, we analyzed the spatial pattern of *A. bungii* in China. Subsequently, we investigated the impacts of climate change and human activities on the distribution of *A. bungii* habitats using the MaxEnt model. Finally, we used the MigClim model, in conjunction with the dispersal capacity, dispersal barriers, and suitable habitat maps of the species, to simulate the potentially occupied suitable *A. bungii* habitat area and size trends due to the species' migratory capacity and dispersal barriers in the future climate model. Our goals were to: (1) identify the most critical factors associated with human activities and climate change that impact the distribution of *A. bungii* in China; (2) to compare the differences in potential habitat distribution and area of *A. bungii* with and without human interference; (3) exploring future changes in distribution patterns of potential *A. bungii* habitats under different climate scenario; (4) to understand the potential distribution area that *A. bungii* can occupy in the future (the diffusion capacity of *A. bungii* and the barriers to diffusion are used as constraints); and (5) to clarify the spatial variations and development trends of *A. bungii*. The results of this study will help to further explain the distribution characteristics and development trend of *A. bungii* in China from an ecological point of view, and provide a scientific basis and reference for local and governmental development of reasonable control strategies.

## 2 Materials and Methods

### 2.1 Data

#### 2.1.1 Species Occurrence Sites

**FIGURE 1:**
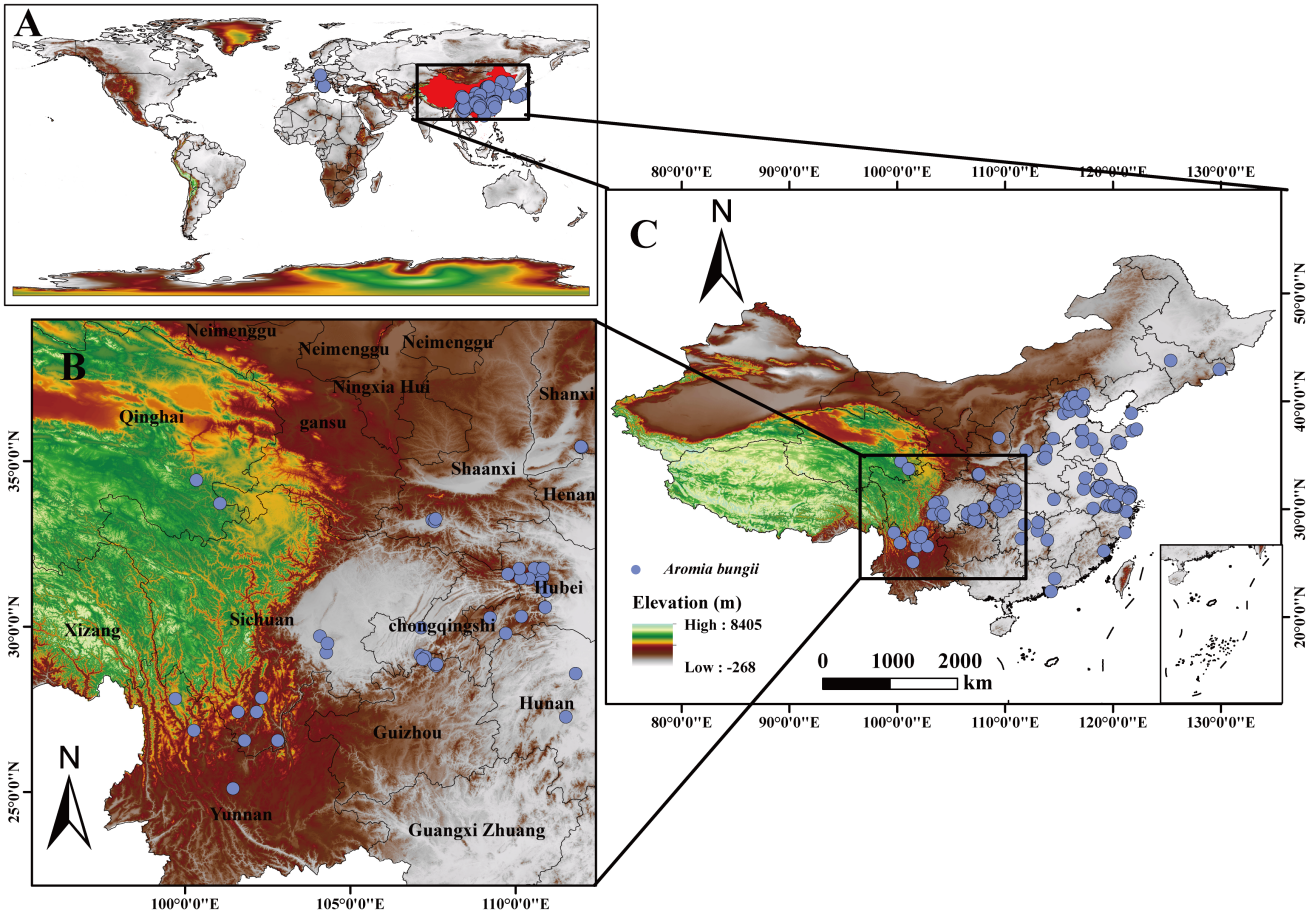
Occurrence records of the *Aromia bungii* population. (A) Records of the occurrence of *A. bungii* worldwide. (B) Field collection of *A. bungii* occurrences. (C) Records of the occurrence of *A. bungii* within China.

**FIGURE 2:**
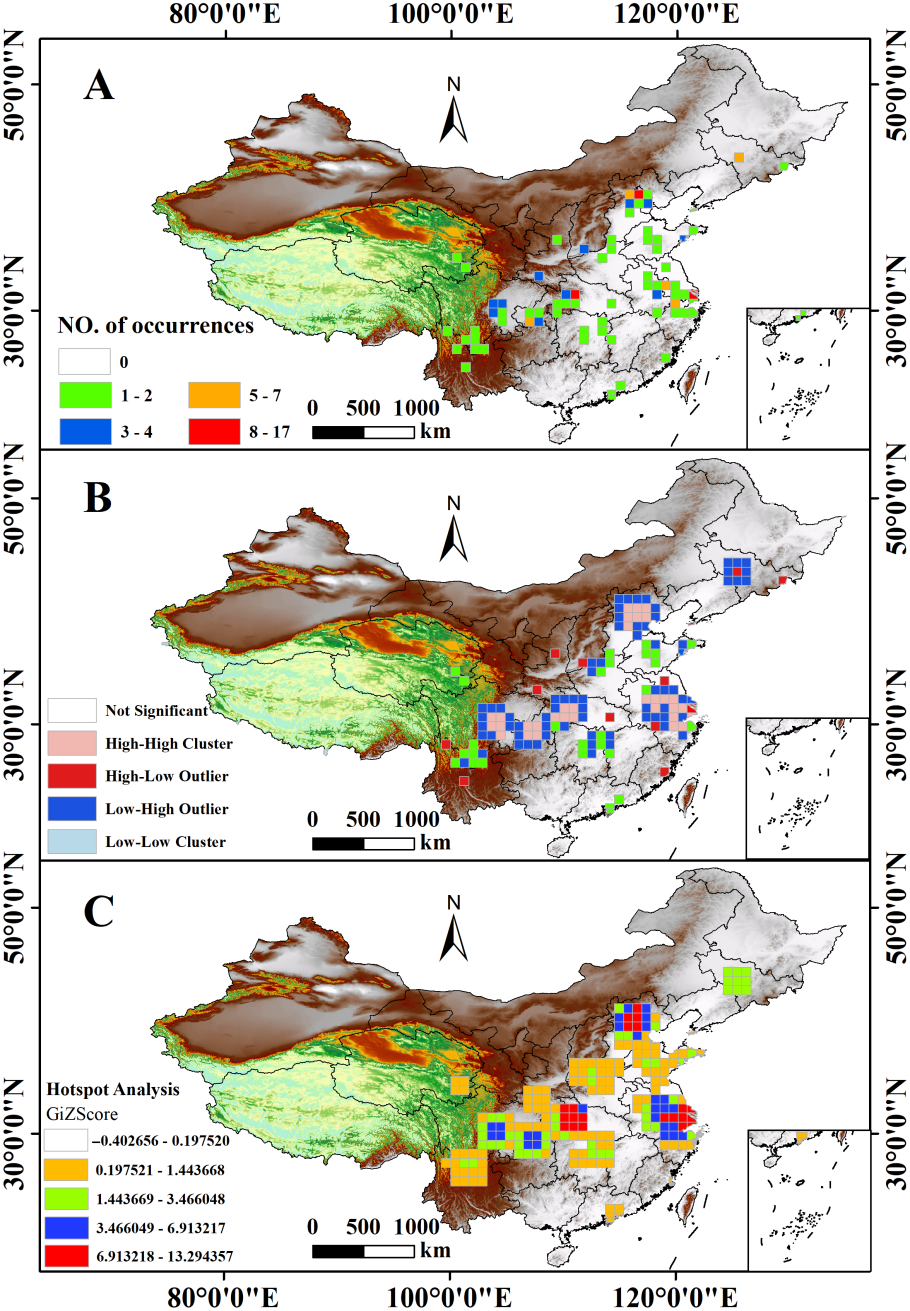
Spatial analysis of *Aromia bungii* (100 × 100 km). (A) Occurrence of *A. bungii* distributed within China. (B) Local Moran's *I* of *A. bungii*. (C) Hot spot analysis of *A. bungii*.

To generate the occurrence records of *A. bungii* used in the modeling, we collected data from various sources (Figure 1), including (1) from 2013 to the present, this experimental group has obtained the latitude, longitude, and altitude of the *A. bungii* occurrence records using GPS in the field surveys conducted in Shaanxi, Shanxi, Qinghai, Anhui, Hubei, Hunan, Guangxi, Chongqing, Sichuan, Yunnan, and other provinces and municipalities to obtain their own data for this study (Figure 1B); (2) book materials and online references (CNKI, https://www.cnki.net/; WOS, https://www.webofscience.com/wos); and (3) two online public databases, the Global Biodiversity Information Facility (GBIF) (https://doi.org/10.15468/dl.bqnzyf, accessed on February 26, 2024) and an iNaturalist (https://www.inaturalist.org/, accessed on February 27, 2024) (Figure 1A). To address the issue of missing coordinates in the dataset, we used Google Earth for location identification. The process involved reviewing all records that were missing precise coordinates and manually locating those locations using Google Earth's capabilities (locating location records at points in administrative units such as district, county, and municipal governments where they occurred) in order to obtain this information. A total of 178 distribution records of *A. bungii* were collected in this study (Figure 1C).

To avoid data duplication, which can lead to overfitting, duplications due to spatial clustering were removed using the “ENMTools” package (version 1.0.4) for the R platform (https://www.r‐project.org/, accessed October 1, 2023) (Warren et al. 2021). The tool automatically adjusts the size of the environmental factor grid used for analysis and removes redundant data within the same grid, which is set to 2.5 arc minutes (~5 km). The method is fast, efficient, and the analysis results are more meaningful. As a result, the occurrences obtained are necessarily smaller than the actual distribution area. Finally, *A. bungii* retained 140 occurrence data points for the construction of the MaxEnt model (Figure 1).

#### 2.1.2 Environmental Variables

In this study, we initially selected 31 environmental variables (Table S1) that may influence the distribution of *A. bungii*, including bioclimatic, topographic, vegetation, solar radiation, and human activity data. The climate data were downloaded from WorldClim version 2.1 (https://www.worldclim.org/, accessed on December 24, 2023), the current period (considering 1970–2000 data as the current dataset), and future climate data (2050s: years 2041–2060 and 2070s: years 2061–2080), which included 19 bioclimatic variables at a resolution of 2.5 arc minutes. For future climate data (2050s and 2070s), we used the BCC‐CSM2‐MR global circulation model under four shared socioeconomic pathways (SSP: SSP1‐2.6, SSP2‐4.5, SSP3‐7.0, and SSP5‐8.5). BCC‐CSM2‐MR performs well in simulating the climate of China and its neighboring regions, and provides relatively accurate climate prediction data, which is suitable for use in this study (Fick and Hijmans 2017). Similarly, elevation data for 2.5 arc minutes were downloaded from WorldClim. Slope and aspect data were extracted using ArcGIS Map 10.8.1 software (https://desktop.arcgis.com/, ESRI, Redlands, CA, USA). The normalized difference vegetation data were downloaded from the Center for Resource and Environmental Sciences and data of the Chinese Academy of Sciences (https://www.resdc.cn/, accessed on January 19, 2024; Qing et al. 2020). In addition, we have downloaded solar radiation data (https://www.ufz.de/gluv/index.php?en=32367; Khatib and Elmenreich 2015). Human activity data were downloaded from socioeconomic data and applications center (https://sedac.ciesin.columbia.edu, accessed on January 15, 2024), including the global human influence index and global human footprint (Yang et al. 2022a, 2022b). World and Chinese administrative division maps were obtained from the standard map service website of the National Bureau of Surveying, Mapping and Geographic Information (http://bzdt.ch.mnr.gov.cn/index.html, accessed on December 9, 2023). Finally, we utilized the “Resampling” and “Extraction” tools within the ArcGIS Map software to standardize the 31 environmental variables into a consistent format (Table S1).

The environmental variables are correlated with each other to avoid the problems of autocorrelation and multicollinearity repetition of some variables, which in turn affects the accuracy of model prediction. In this article, we first used the Jackknife method to obtain the contribution rates of 31 environmental variables in the MaxEnt model (https://biodiversityinformatics.amnh.org/open_source/maxent/; Table S1; Shcheglovitova and Anderson 2013). And then we used the “ENMTools” package in R software to conduct Pearson's correlation analysis on the 31 environmental variables, and selected the variables with correlation coefficients |*r*| ≤ 0.9 for modeling, and when the correlation coefficient |*r*| > 0.9 between two environmental variables, the variables with larger contribution rates were selected to be input into the model (Figure S1; Warren et al. 2021). Finally, 13 bioclimatic factors were retained for import into the MaxEnt model.

### 2.2 Geospatial Autocorrelation Analysis

The Moran's *I* and Getis‐Ord Gi* methods are considered complementary tools and are among the most commonly used spatial hot spot detection methods (Zhai et al. 2023). The “spatial statistics tool” in ArcGIS Map software was used to calculate the global Moran's *I* index and the Getis‐Ord General *G* index for *A. bungii* in China (100 × 100 km). The purpose of this study was to determine whether the distribution pattern of *A. bungii* in China is clustered or random. Using the Getis‐Ord Gi* method in the “Hot spot analysis,” we were able to study the clustered areas of *A. bungii* more precisely. This approach assesses spatial clusters with significantly high or low values relative to the mean value of occurrences by contrasting the mean values of local and global areas (Zha et al. 2024).

#### 2.2.1 Global Spatial Autocorrelation

The global spatial autocorrelation parameters include the global Moran's *I* index and the Getis‐Ord General *G* index. The two parameters are a combination of data locations and attribute values to determine autocorrelation between spaces and are used to detect spatial clusters and their trends (Zhang et al. 2008).

The global Moran's *I* index for spatial autocorrelation is calculated as follows:

$$I=\frac{n}{S_{0}}\frac{\sum_{i=1}^{n}w_{i,j}z_{i}z_{j}}{\sum_{i=1}^{n}z_{i}^{2}}$$

where *Z*_*i*_ is the deviation of the attribute of *A. bungii* from its mean value (*x*_*i*_ − $\overset{⃐}{x}$) and *W*_*ij*_ is the aggregate of all spatial weights. If pixel *i* and pixel *j* are adjacent, the value of the corresponding element in the matrix *W*_*ij*_ is 1; otherwise, it is 0. *n* is the number and is equal to the total number of *A. bungii*, *S*_*0*_ is an aggregation of all spatial weights.

$$S_{0}=\sum_{i=1}^{n}\sum_{j=1}^{n}w_{i,j}$$

To investigate the statistical significance of the Moran's *I* statistic, Z(*I*) is calculated as follows:

$$Z\left(I\right)=\frac{1-E\left(I\right)}{\sqrt{\mathrm{Var}\left(I\right)}}$$

$$E\left(I\right)=-1/\left(n-1\right)$$

$$V\left(I\right)=E\left(I^{2}\right)-E{\left(I\right)}^{2}$$

where *E*(*I*) is the expected value of *I*: *E*(*I*) = −1/(*n* − 1), and $\sqrt{\mathrm{Var}\left(I\right)}$ is the expected variance of *I*: $\sqrt{\mathrm{Var}\left(I\right)}=E\left(I^{2}\right)-E{\left(I\right)}^{2}$. When a significance level is established, a Moran's *I* approaching +1 indicates that the *A. bungii* data are spatially correlative. The global Moran's *I* index combines data location and attribute values to determine autocorrelation between spaces. A value of 0 < Moran's *I* ≤ 1 indicates positive spatial correlation, with larger values indicating stronger correlation. Conversely, −1 ≤ Moran's *I* < 0 indicates negative spatial correlation, with smaller values indicating greater spatial difference. When Moran's *I* = 0, it indicates spatial randomness.

The Getis‐Ord General *G* index is determined as follows (Ren, Shang, and Zhang 2020):

$$G=\frac{\sum_{i=1}^{n}\sum_{j=1}^{n}W_{\mathrm{ij}}x_{i}x_{j}}{\sum_{i=1}^{n}\sum_{j=1}^{n}x_{i}x_{j}}$$

where *x*_*i*_ is *A. bungii* of pixel *i*, *x*_*j*_ is *A. bungii* of pixel *j*, and *W*_*ij*_ is the spatial weight inversely correlated with the distance between the two locations. The expectation of *G* and *Z*(*I*) is calculated as follows:

$$\begin{array}{rr} E\left(G\right) & =\frac{\sum_{i=1}^{n}\sum_{j=1}^{n}W_{\mathrm{ij}}}{n\left(n-1\right)}\\ Z\left(I\right) & =\frac{G-E\left(G\right)}{\sqrt{\mathrm{Var}\left(G\right)}} \end{array}$$

In general, if the value of *G* is greater than *E*(*G*), the high‐value data tend to cluster. Otherwise, low‐value data tend to cluster. The *A. bungii* data in the regions are distributed randomly when *G* is equal to *E*(*G*).

#### 2.2.2 Analysis of Hot Spots (Getis‐Ord Gi*) of *A. bungii* in China

The Getis‐Ord Gi* is a spatial statistical approach for describing and visualizing the spatial distributions of ecosystem services, discovering local patterns of spatial associations, identifying heterogeneous units, and suggesting spatial regimes. Compared with the traditional approach of identifying process‐related zones, Getis‐Ord Gi* has been shown to be effective in identifying cluster‐related heterogeneous units (Guerri et al. 2022). The Getis‐Ord Gi* was used to identify hot spots with statistical significance, and the extremes and hot spots were spatially superimposed to explore the clustering characteristics of the *A. bungii* distribution in China (100 × 100 km).

The Getis‐Ord Gi* is calculated as follows:

$$z\left(G_{i}^{*}\right)=\frac{\sum_{j=1}^{n}w_{\mathrm{ij}}\left(d\right)x_{j}-\bar{x}w_{i}^{*}}{\sqrt{\frac{s^{2}}{n-1}\left(n\left(\sum_{j=1}^{n}w_{\mathrm{ij}}^{2}\left(d\right)\right)-w_{i}^{*2}\right)}}$$

$$S=\sqrt{\frac{\sum \sum_{i}{\left(x_{i}x_{j}\right)}^{2}}{n}-{\bar{x}}^{2}},\;{\bar{x}}^{2}=\sum_{j}x_{j}/n$$

$$w_{i}^{*}=\sum_{j}W_{\mathrm{ij}}\left(d\right)$$

where *x*_*j*_ is the attribute value of element *j*, *w*_*i,j*_(*d*) is the spatial weight between elements *i* and *j* and is the *n × p* element of the spatial weight matrix *w*, and *n* is the total number of elements. The matrix *w* is derived from the threshold distance *d* between *x*_*i*_ and *x*_*j*_. Threshold distance *d* is defined by the rule that all elements within this distance are called neighbors, and the weights between these elements take the value of 1 in the matrix *w*, while elements not within this distance take the value of 0 in the matrix *w*. For the sake of uniformity in the calculation, the spatial weights formed by the rule of neighbors are converted into distance weights to participate in the *G*_i_* measure, which calculates a spatial weight centered on the *i*th position, whose value is equal to the value of its neighbors with respect to the spatial weights. The sum of the products of the values of the neighbors and the spatial weights is equal to the sum of all the data values. The identification of aggregation regions for *A. bungii* involves analyzing the *G*_i_* *z* scores. Elevated *z* scores signify densely clustered high values, characterizing “hot spot” regions, whereas diminished *z* scores indicate densely clustered low values, defining “cool spot” regions.

### 2.3 Optimization of the MaxEnt Model Parameters

Regularized multipliers (RMs) and feature combinations (FCs) serve as crucial parameters within the MaxEnt model, and optimizing these parameters can substantially enhance the model's accuracy (Radosavljevic, Anderson, and Araújo 2014). FCs encompass five feature types, denoted as L (linear), Q (quadratic), H (hinge), P (product), and T (threshold). The adjustment of the RMs and FCs parameters was carried out utilizing the “ENMeval” package within R software. Initially, RMs were configured from 0 to 4 at intervals of 0.5 based on the preserved distribution data and environmental variables. Six FCs were designated to identify the optimal parameter combinations: L, LQ, H, LQH, LQHP, and LQHPT. We constructed a total of 48 different combined RMs and fc models and selected the model with the smallest delta. AICc value as the optimal model (Figure S2). Finally, under natural environmental variable conditions, RMs were set to 2 with the FCs designated “LQHP” (Figure S2A); under conditions incorporating both natural environment variables and human activities, RMs were set to 0.5 with the FCs specified “LQ” (Figure S2B). The additional parameter settings for the optimal model were as follows: 25% of the distribution points for each species were selected as the test set, while 75% were used for training. The maximum number of iterations was set to 5000, the maximum number of background points was limited to 10,000, and the repetitions were carried out 10 times. The closer the test omission rate is to the theoretical omission rate, the higher the accuracy of the model construction. The importance of each variable was measured using the Jackknife method, and response curves were generated (Campos et al. 2023; Lin et al. 2024).

### 2.4 MaxEnt Model Analysis

#### 2.4.1 MaxEnt Model Evaluation and Validation

This study utilized receiver operating characteristic (ROC) analysis, where the area under the ROC curve (AUC) value was used to evaluate the accuracy of the model results (Phillips, Anderson, and Schapire 2006). The AUC ranges from 0 to 1, with higher values indicating better model performance. The AUCs used to assess the accuracy of the MaxEnt prediction results are divided into five levels: when 0 < AUC ≤ 0.6, the prediction results were divided into five levels: 0 < AUC ≤ 0.6, “failure”; 0.6 < AUC ≤ 0.7, “poor”; 0.7 < AUC ≤ 0.8, “general”; 0.8 < AUC ≤ 0.9, “good”; and 0.9 < AUC ≤ 1.0, “excellent.”

#### 2.4.2 Changes in the Potential Distribution Areas of *A. bungii*

Three models were developed to investigate the influence of both natural environmental factors and human activities on the geospatial distribution patterns of *A. bungii*: (1) prediction of natural environmental factors (bioclimate + topography + solar radiation + the normalized difference vegetation index) under the current climate model; (2) prediction of natural environmental factors (bioclimate + topography + solar radiation + the normalized difference vegetation index) + human activity (global human footprint + global human influence index) under the current climate model; and (3) prediction of natural environmental factors (future bioclimate + topography + solar radiation + the normalized difference vegetation index) under the future climate model. Among them, models (1) and (2) are based on current climate model projections, while model (3) is based on future climate model projections. Models (1) and (2) analyze the effects of human activities on the suitable habitat of *A. bungii*, while models (1) and (3) analyze the effects of climate change on the suitable habitat of *A. bungii* (Xu et al. 2024).

In this study, the average value after 10 repetitions of the optimal MaxEnt model was used as the final result, based on the logical value of the probability of existence (*p*) of the species, and the final result was converted to raster form and visualized by using ArcGIS Map, and the area proportion of each type of suitable habitat was calculated (Low et al. 2021; Campos et al. 2023). Finally, ArcGIS Map and GraphPad Prism 9.0 software were used to construct the graphs.

The suitability results for *A. bungii* were categorized into four groups based on the natural breakpoint method in ArcGIS Map software: unsuitable suitability habitat (*p* < 0.093677), low suitability habitat (0.093677 ≤ *p* < 0.262295), moderately suitable habitat (0.262295 ≤ *p* < 0.468384), and highly suitable habitat (0.468384 ≤ *p* < 1). For comparison, both current and future climate models were categorized using the same thresholds.

#### 2.4.3 Dynamics of *A. bungii* Under Future Climate Scenarios

The “Distribution Changes between Species Distribution Models (SDMs)” function in SDMToolbox v2.6 was used to investigate the dynamic changes of *A. bungii* under future climate scenarios (Tan et al. 2019). As a result, four outputs were obtained: “expansion,” “no occupancy,” “unchanged,” and “contraction.” In addition, multivariate environmental similarity surface (MESS) was used to analyze the extent to which climate change and its main driving variables affected the potential suitability areas of *A. bungii* in China (Nori et al. 2011). The environmental variables of *A. bungii* contemporary potential fitness areas were used as reference layers, and the similarity between them was calculated for current and future climates. The similarity value (S) reflects the degree of similarity between the climatic conditions of a point and those of the reference layer during a specific period of time. Negative values indicate that at least one of the values of the environmental variables at the point is out of the range of the corresponding value of the reference layer, which is called a climatic anomaly, while the maximum value of 100 means that the climate at the point is completely normal. This operation is realized by running the “density.tools.Novel” tool in the “maxent.jar” file in the command window, and the ASCII file exported from the model were imported into ArcGIS Map 10.8.1 software for drawing.

#### 2.4.4 Change of Potential Distribution Center Shift Under Future Climate Scenarios

The potential distribution centers of the areas of *A. bungii* with a probability (*p*) of existence greater than 0.093677 were calculated for each future period using the “Centroid Changes (Lines)” tool in SDMToolbox v2.6 (Mkala et al. 2022). The locations where the potential distribution centers shifted were then compared for different scenarios in the modern and future periods (including four different carbon emission scenarios). Line segments were used to connect the calculated potential distribution center points, creating shift routes that reflect the spatial shift routes of the main suitable areas of *A. bungii* under future climate conditions.

### 2.5 MigClim Model Analysis

With reference to the method of Wang et al. (2019), the barrier diffusion simulations were carried out with the aid of the “MigClim” package in R software. Other parameters, including initial species distribution, habitat suitability map, dispersal barriers, propagule production potential, minimum and maximum distances for LDD, and number of environmental changes and dispersal steps, were used in the MigClim model as suggested by Engler and Guisan (2009). The dispersal potential of *A. bungii* was estimated for the years 2050 and 2070. The MigClim output raster files were reclassified as “Barriers and unsuitable areas,” “Occupied habitats,” and “Unoccupied habitats” based on the status of the raster cells.

## 3 Results

### 3.1 Geospatial Pattern Autocorrelation Analysis of *A. bungii* in the Current Period

The spatial correlation and aggregation status of *A. bungii* were analyzed using ArcGIS Map software. First, the global Moran's *I* index is 0.161080 (*z* score = 13.715704, *p* < 0.05) (Table S2), which indicates that there is 99% confidence that the distribution of *A. bungii* is not random and that the probability of a random distribution is less than 1%. Thus, the distribution exhibits a positive spatial correlation. The Getis‐Ord General *G* index was 0.044963 (*z* score = 13.862348, *p* < 0.05) (Table S3), indicating that the probability of the *A. bungii* distribution pattern randomly generating a high clustering pattern was less than 1%, and the distribution clustered in regions with high values.

The quantity of *A. bungii* was determined by means of a fishing net measuring 100 × 100 km. The results showed that the Beijing, Shanghai, and Shennongjia forestry districts in Hubei had the highest number of *A. bungii*, while the northwestern and northeastern regions had the lowest number of *A. bungii* (Figure 2A). Local Moran's *I* autocorrelation analysis can directly show the local spatial aggregation or discrete areas of *A. bungii*. High‐value areas of *A. bungii* are in the southwestern part of the study area, are concentrated in Beijing, Jiangsu, Anhui, Zhejiang, Hubei, Chongqing, and Sichuan provinces and municipalities, are at serious risk of infestation, and should be strengthened in the prevention and treatment of these areas (Figure 2B). In addition, the Getis‐OrdGi* method identified a significant number of hot spots (i.e., high‐density areas surrounded by high‐density areas) in China, specifically in Hebei, Jiangsu, Shanghai, Anhui, Zhejiang, and Hubei (Figure 2C).

### 3.2 Evaluation the Accuracy of Predictions From Optimized Species Distribution Models

The simulated predictions were evaluated by optimizing the average AUC value of the 10 outcomes in the MaxEnt model (Table 1). The average training AUC value for the model with natural environmental variables was 0.9429, and the average test AUC value was 0.9178. Similarly, the average training AUC value for the model with natural environmental variables and human activity was 0.9494, and the average test AUC value was 0.9312. All average training AUC and average test AUC were greater than 0.9 under the four different future carbon emission scenarios (Table 1). The results demonstrate that the optimized MaxEnt model is both more accurate and more precise.

**TABLE 1:** Model accuracy evaluation.

Shared socioeconomic pathways	Train AUC (avg)	Test AUC (avg)
Current environmental variables	0.9429	0.9178
Current environmental variables + Human activity	0.9494	0.9312
Future‐SSP1‐2.6 2040‐2060	0.9411	0.9069
Future‐SSP1‐2.6 2060‐2080	0.9402	0.9212
Future‐SSP2‐4.5 2040‐2060	0.9443	0.9193
Future‐SSP2‐4.5 2060‐2080	0.9439	0.9126
Future‐SSP3‐7.0 2040‐2060	0.9385	0.9213
Future‐SSP3‐7.0 2060‐2080	0.9450	0.9167
Future‐SSP5‐8.5 2040‐2060	0.9443	0.9291
Future‐SSP5‐8.5 2060‐2080	0.9445	0.9301

### 3.3 *Aromia bungii* in Relation to Environmental Variables

**FIGURE 3:**
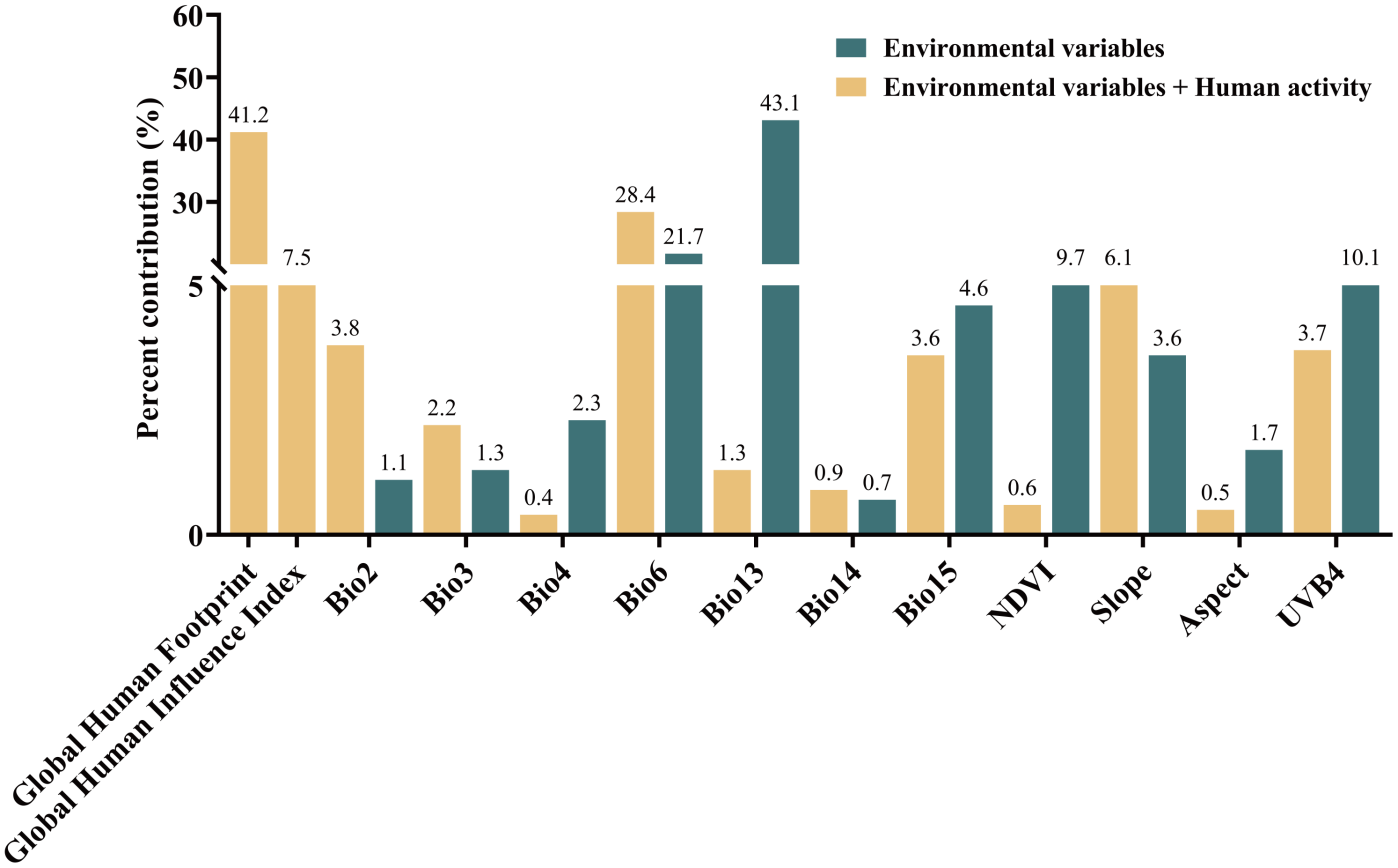
The contribution rates of the environmental variables in MaxEnt model.

This study identified the primary environmental factors that influence the geographic distribution of *A. bungii* (Figure 3). The MaxEnt model outputs and the response curves based on the Jackknife method were used to determine these factors. The potential geographic distribution of *A. bungii* under the influence of the natural environment is affected by various factors. The MaxEnt model predictions indicated that Bio13 (43.1%), Bio6 (21.7%), Bio27 (10.1%), and Bio21 (9.7%) had cumulative contributions of 84.6% (Figure 3). Considering the impact of human activities, the primary variables affecting the potential geographic distribution of *A. bungii* were Bio30 (41.2%), Bio6 (28.4%), Bio31 (7.5%), and Bio22 (6.1%), with a cumulative contribution of 83.2% (Figure 3). With the inclusion of anthropogenic factors, the variables that showed the greatest changes in contribution were Bio13 (41.8% increase), Bio21 (9.1% decrease), Bio6 (6.7% decrease), and Bio27 (6.4% increase) (Figure 3). This suggests that precipitation, the normalized difference vegetation index, temperature, solar radiation, and human activities are the primary factors influencing *A. bungii*.

**FIGURE 4:**
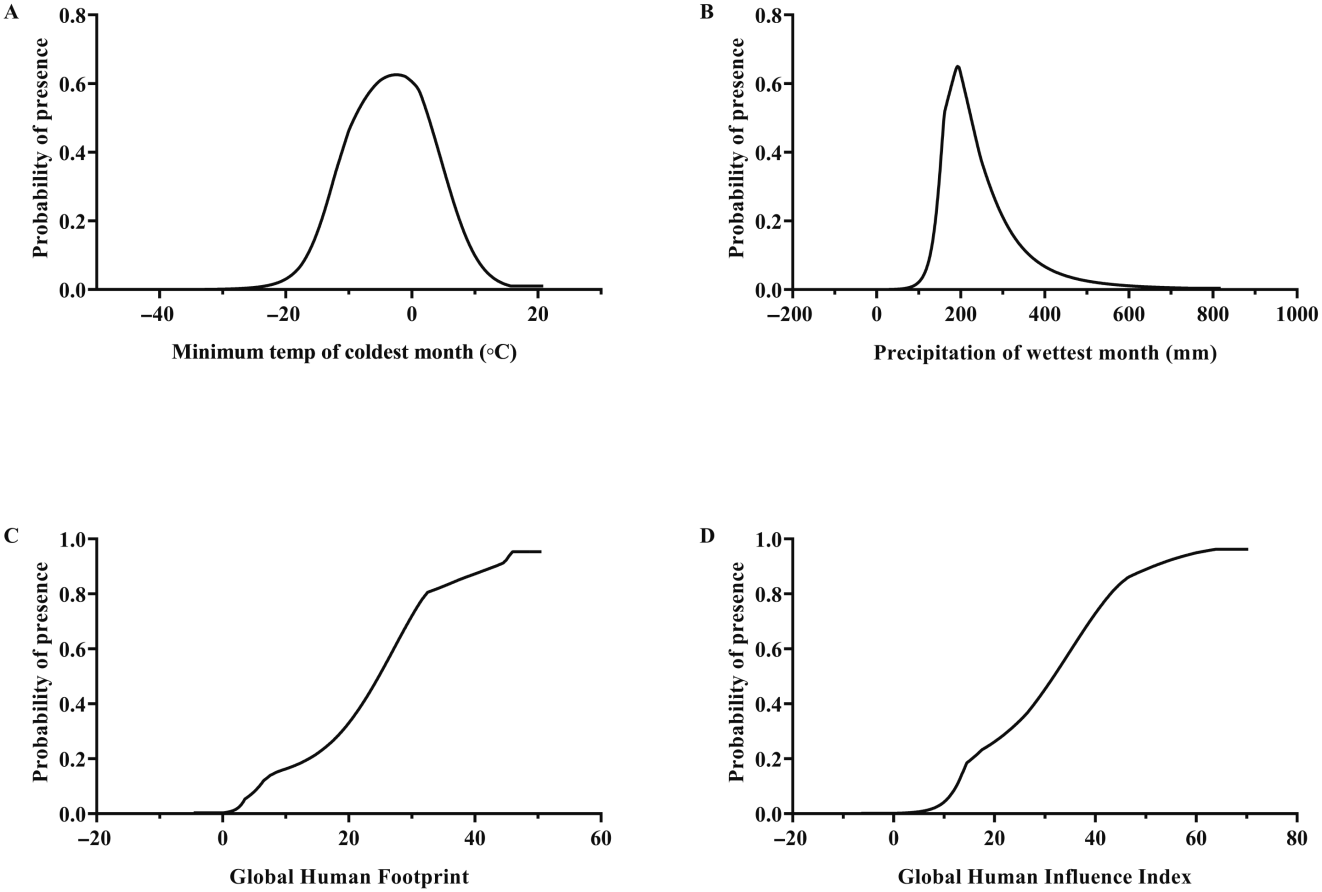
Impact of the most important environmental variables on *Aromia bungii*. (A) Bio6, minimum temperature of coldest month. (B) Bio13, precipitation of wettest month. (C) Bio30, global human footprint. (D) Bio31, global human influence index.

The response curves between the probability of the presence of *A. bungii* and several important environmental variables affecting its distribution in this study are shown in Figure 4. The survival probability of *A. bungii* was positively correlated with the global human footprint (Bio30) and global human influence index (Bio31). However, the survival probability of *A. bungii* increased and then decreased as the minimum temperature of the coldest month (Bio6) and the precipitation of the wettest month (Bio13) increased. The highest values were observed at −2.41°C and 192.36 mm, with survival probabilities of 62.56% and 64.98%, respectively (Figure 4).

### 3.4 Prediction of the Potential Distribution of *A. bungii* Under Climate and Human Interference in the Current Time Period

**FIGURE 5:**
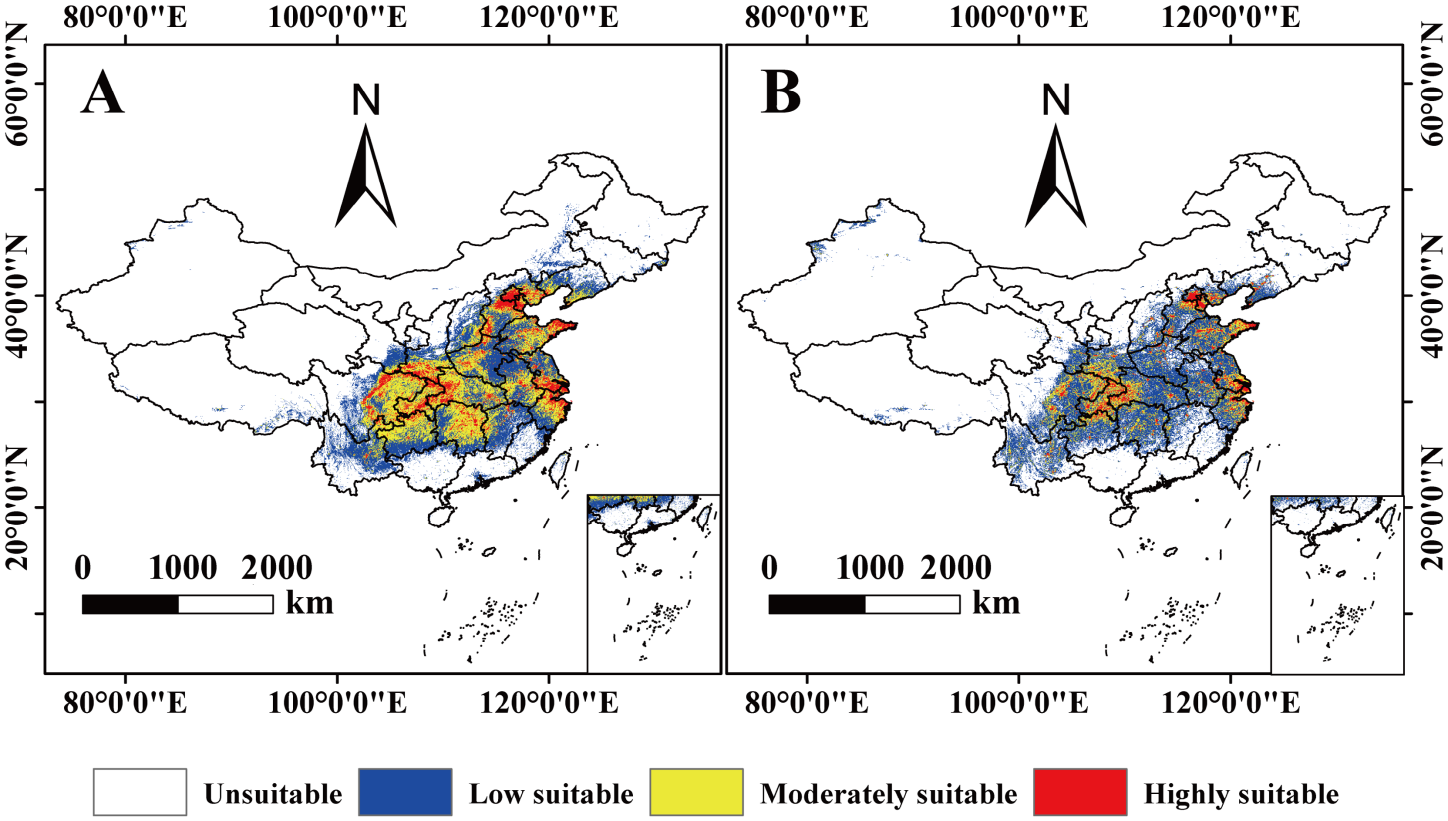
Distribution of suitable habitat for *Aromia bungii* with (A) and without (B) human activity under current climate models.

**FIGURE 6:**
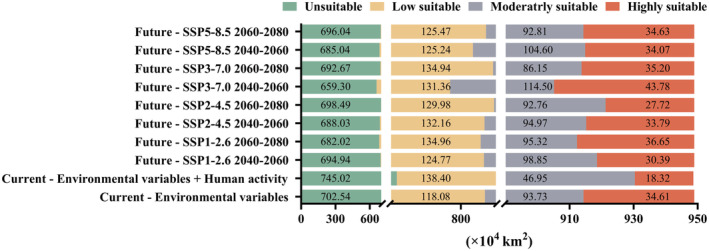
Suitable habitat area for *Aromia bungii* under different climate scenarios.

The MaxEnt model was used to predict the distribution area and size of *A. bungii* with and without human activity disturbance, respectively (Figure 5). Under the influence of natural environmental conditions, the total suitable habitat of *A. bungii* in China is 246.41 × 10^4^ km^2^, mainly concentrated in the central and eastern provinces and municipalities, including Beijing, Tianjin, Hebei, Shanxi, Shandong, Henan, Shaanxi, Jiangsu, Shanghai, Anhui, Zhejiang, Hubei, Hunan, Chongqing, and Sichuan (Figure 5A). The area of highly suitable habitat was 34.61 × 10^4^ km^2^, accounting for 3.65% of China's total area (Table S4). The area of moderately suitable habitat was 93.73 × 10^4^ km^2^, accounting for 9.88% of China's total area (Figure 6). The area of low suitable habitat is 118.08 × 10^4^ km^2^, accounting for 12.44% of the total area of China. The area of unsuitable habitat is 702.54 × 10^4^ km^2^, accounting for 74.03% of China's total area (Table S4).

With the addition of human activity interference, the total habitat area of *A. bungii* in China was 203.67 × 10^4^ km^2^ and was mainly distributed in Beijing, Tianjin, Hebei, Shanxi, Shandong, Henan, Jiangsu, Shanghai, Anhui, Hubei, Hunan, Chongqing, and Sichuan (Figures 5B and 6). The total area of suitable habitat for *A. bungii* decreased by 42.74 × 10^4^ km^2^, compared to the area affected by environmental factors alone, accounting for 4.50% of the total area in China (Table S4). The area of highly suitable habitat was 18.32 × 10^4^ km^2^, accounting for 1.93% of the total area in China. The area of moderately suitable habitat was 46.95 × 10^4^ km^2^, accounting for 4.95% of China's total area. The area of low suitable habitat was 138.40 × 10^4^ km^2^, accounting for 14.58% of China's total area (Figure 6; Table S4).

### 3.5 Prediction of the Potential Distribution of *A. bungii* Under Different Climate Scenarios in the Future

**FIGURE 7:**
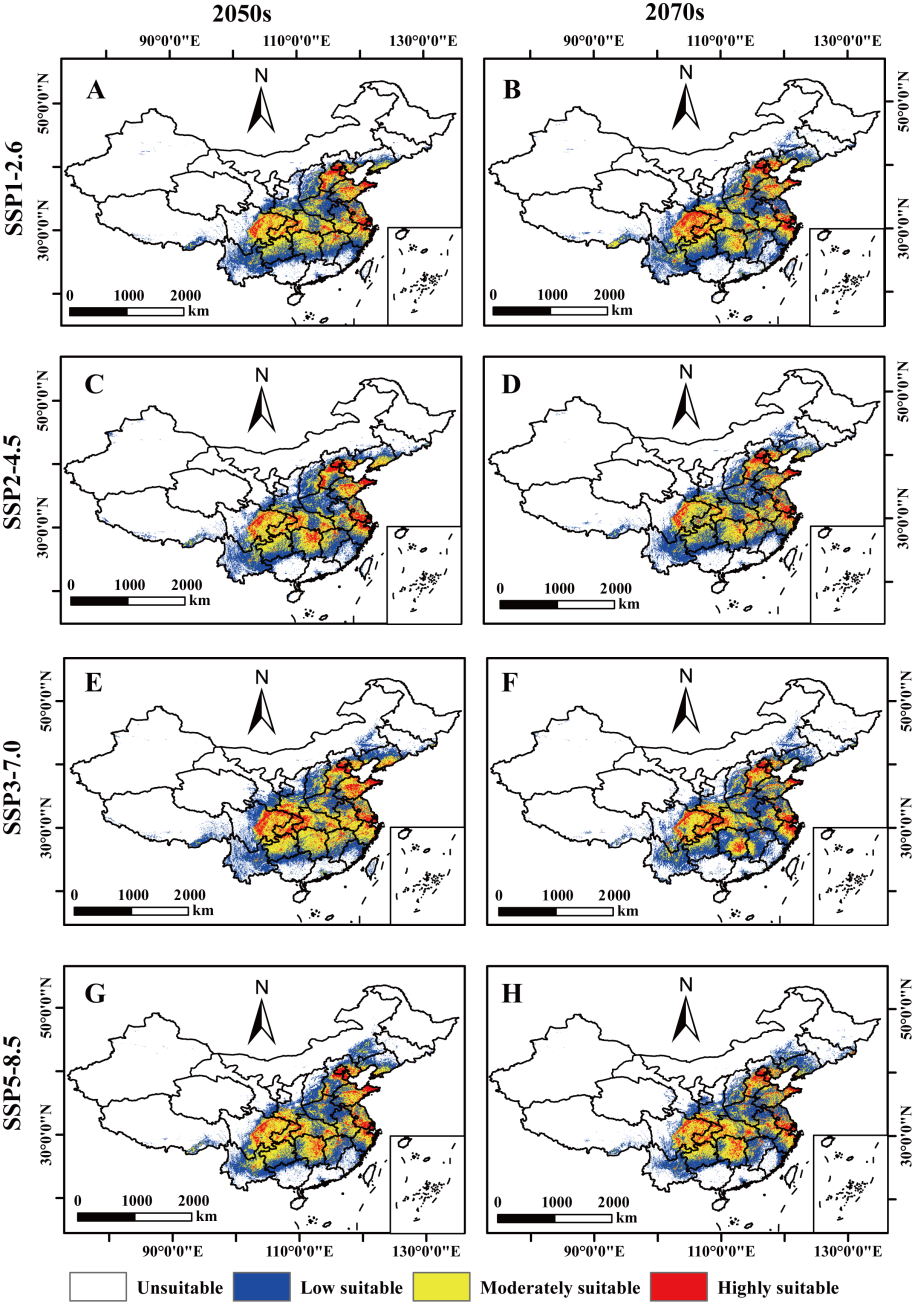
Potential suitable distribution areas for *Aromia bungii* in China under future climate scenarios. (A) SSP1‐2.5‐2050s; (B) SSP1‐2.5‐2070s; (C) SSP2‐4.5‐2050s; (D) SSP2‐4.5‐2070s; (E) SSP3‐7.0‐2050s; (F) SSP3‐7.0‐2070s; (G) SSP5‐8.5‐2050s; (H) SSP5‐8.5‐2070s.

Under different future climate scenarios, the range of suitable habitats for *A. bungii* is predicted to be generally consistent with the current climate scenario, and to be mainly distributed in the areas of Beijing, Tianjin, southern Hebei, Shanxi, central and eastern Shandong, Henan, southern Shaanxi, southern Jiangsu, Shanghai, northern Zhejiang, western Hubei, central Hunan, Chongqing, eastern Sichuan, and northwestern Guizhou (Figure 7). The area of *A. bungii* suitable habitat under the projected future climate scenarios is projected to be 252.92 × 10^4^ km^2^ to 289.65 × 10^4^ km^2^, accounting for 26.65% to 30.52% of China's total area (Figure 6; Table S4); with the largest area of *A. bungii* suitable habitat predicted under the SSP3‐7.0‐2050s and the smallest area of *A. bungii* suitable habitat predicted under the SSP5‐8.5‐2070s (Figure 7).

The analysis of suitable area size shows that under three future climate scenarios, SSP2‐4.5 (2050s: 260.92 × 10^4^ km^2^; 2070s: 250.46 × 10^4^ km^2^), SSP3‐7.0 (2050s: 289.65 × 10^4^ km^2^; 2070s: 256.28 × 10^4^ km^2^), and SSP5‐8.5 (2050s: 263.92 × 10^4^ km^2^; 2070s: 252.92 × 10^4^ km^2^), the area of suitable habitat for *A. bungii* showed a trend of increasing and then decreasing over time compared to the current period (246.41 × 10^4^ km^2^) (Figures 6 and 7). In contrast, for the SSP1‐2.6 pathway (2050s: 254.01 × 10^4^ km^2^; 2070s: 266.94 × 10^4^ km^2^), the area of suitable habitat tended to decrease and then increase (Figure 6; Table S4). The most pronounced change was in the SSP3‐7.0 pathway, followed by the SSP5‐8.5 pathway, while the change was relatively smooth in the SSP1‐2.6 and SSP2‐4.5 pathways (Figures 6 and 7).

### 3.6 Relative Changes in the Potential Distribution Area of *A. bungii* Under Future Climate Scenarios

**FIGURE 8:**
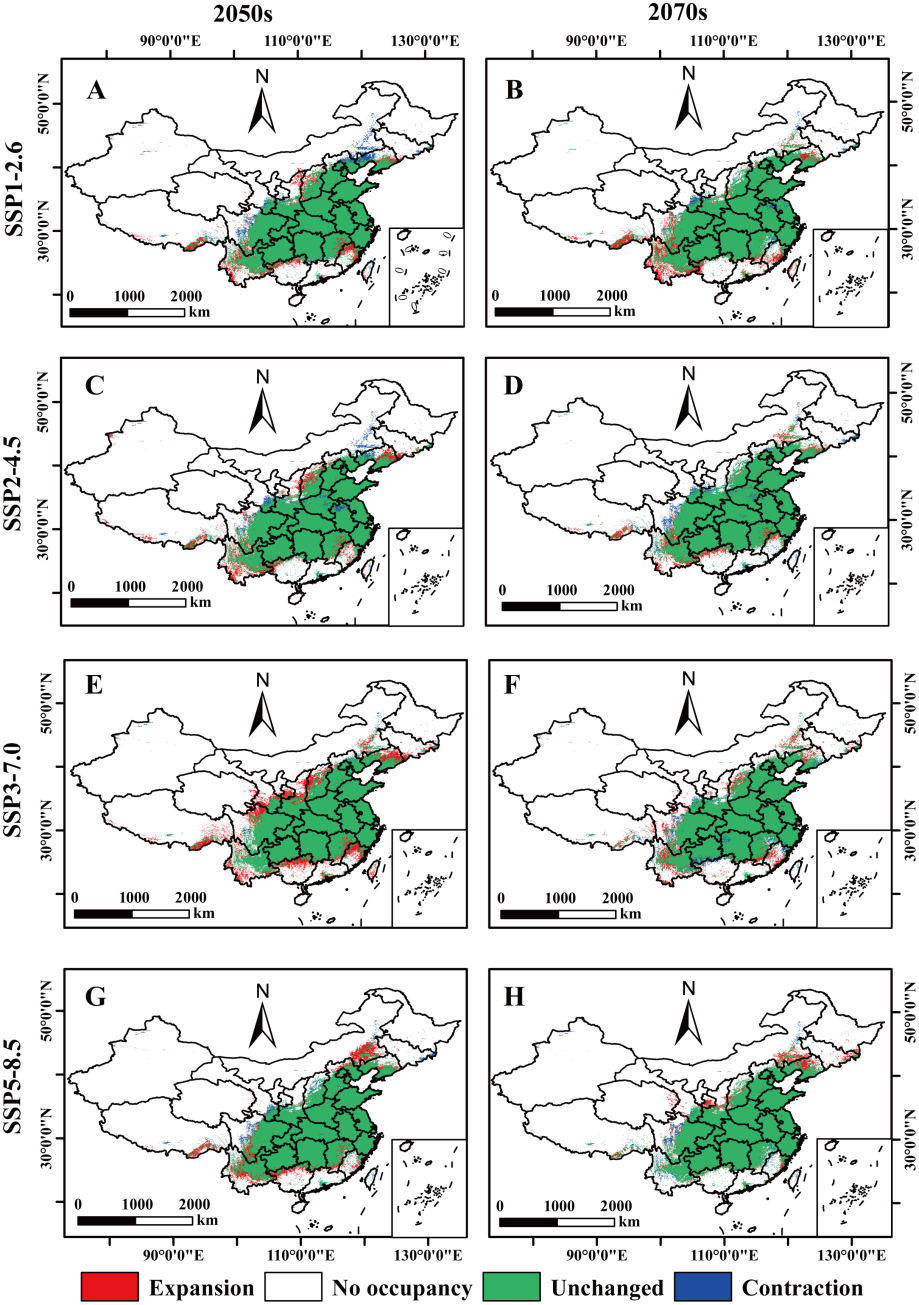
Changes in the distribution of potential habitats of *Aromia bungii* under future climate scenarios. (A) SSP1‐2.5‐2050s; (B) SSP1‐2.5‐2070s; (C) SSP2‐4.5‐2050s; (D) SSP2‐4.5‐2070s; (E) SSP3‐7.0‐2050s; (F) SSP3‐7.0‐2070s; (G) SSP5‐8.5‐2050s; (H) SSP5‐8.5‐2070s.

The relative changes in the potential distribution of *A. bungii* were obtained by analyzing the differences between the current and future distribution strata (Figure 8). The results showed that the expansion area of *A. bungii* was consistently larger than the contraction area in the future climate scenarios, which predicted that the suitable habitat of *A. bungii* would increase (Table S5). The projected area of expansion ranges from 18.16 × 10^4^ km^2^ to 29.82 × 10^4^ km^2^, and the contraction area is predicted to be in the range of 6.71 × 10^4^ km^2^ to 14.39 × 10^4^ km^2^ (Table S5). The expansion occurred mainly in Liaoning, Shanxi, Shaanxi, Jiangxi, Fujian, Taiwan Island, Guangxi, Yunnan, and Xizang. The contractions were mainly in Jilin, Liaoning, Inner Mongolia, Hebei, Shaanxi, Gansu, Jiangxi, Hunan, Taiwan, Sichuan, and Xizang (Figure 8). The expansion area of *A. bungii* under SSP3‐7.0‐2050s is the largest, at 48.70 × 10^4^ km^2^, and the expansion area under SSP2‐4.5‐2070s is the smallest, at 18.16 × 10^4^ km^2^.

**FIGURE 9:**
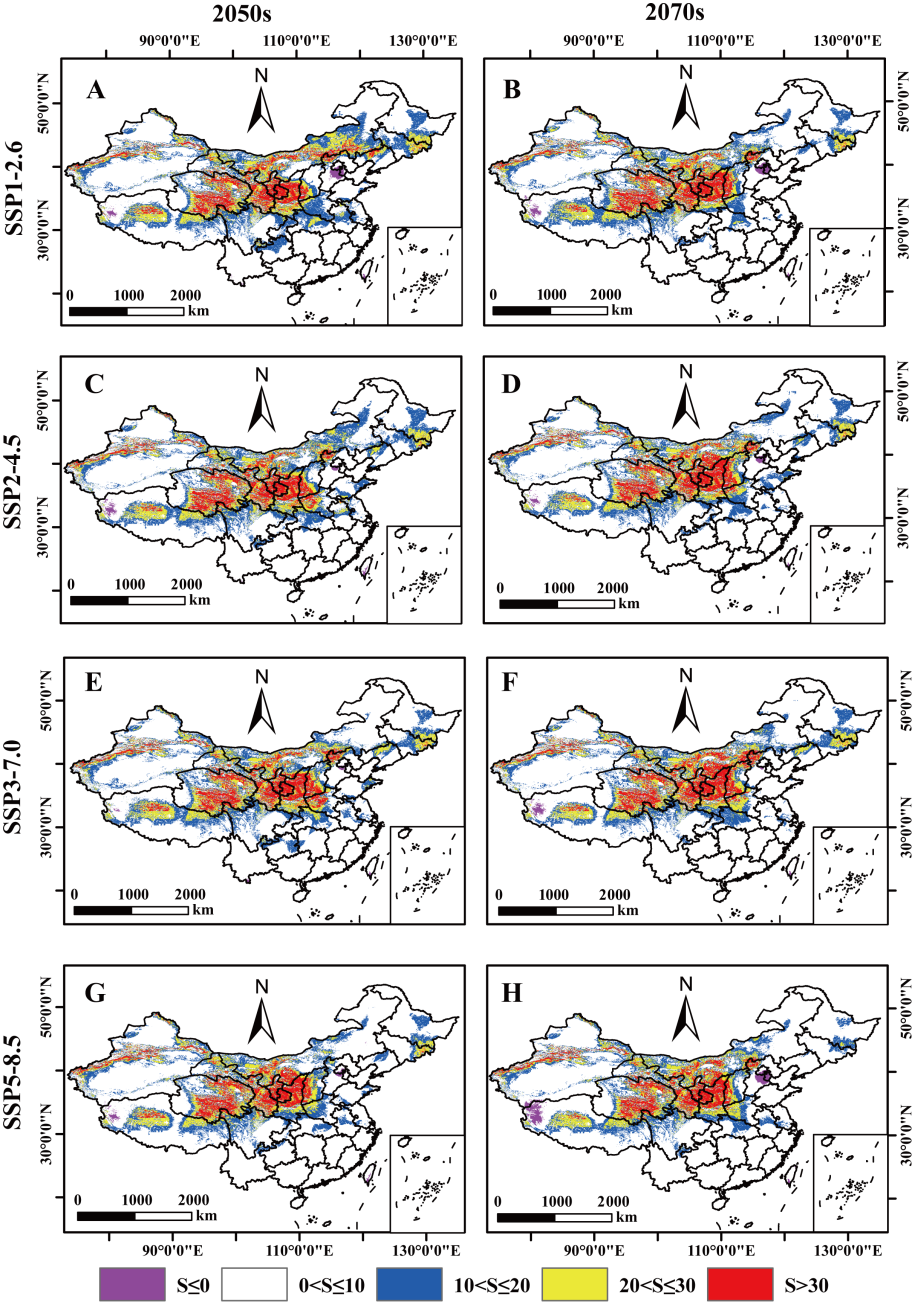
Multivariate environmental similarity surface (MESS) analysis for *Aromia bungii* under future climate scenarios. (A) SSP1‐2.5‐2050s; (B) SSP1‐2.5‐2070s; (C) SSP2‐4.5‐2050s; (D) SSP2‐4.5‐2070s; (E) SSP3‐7.0‐2050s; (F) SSP3‐7.0‐2070s; (G) SSP5‐8.5‐2050s; (H) SSP5‐8.5‐2070s.

In addition, the MESS analyses indicate that the area of *A. bungii* climate anomalies is likely to decrease in the 2050s and 2070s under three climate scenarios, namely SSP1‐2.6, SSP3‐7.0, and SSP5‐8.5. Specifically, the area of *S* values decreases over time, as demonstrated by the temporal correlation between the aforementioned scenarios (Figure 9; Table S6). However, the area of climatic anomalies under the SSP2‐4.5 pathway exhibited an increasing trend. Furthermore, under future climate scenarios, Inner Mongolia, Shanxi, Ningxia Hui Autonomous Region, Gansu, Qinghai, and Shaanxi exhibit a high degree of similarity with the climatic conditions of their origins (Figure 9). This indicates that the climatic zones of the potential distribution of *A. bungii* differ significantly from the reference layer.

### 3.7 Potential Distribution Center Shifts of *A. bungii* Under Different Scenarios in the Future

**FIGURE 10:**
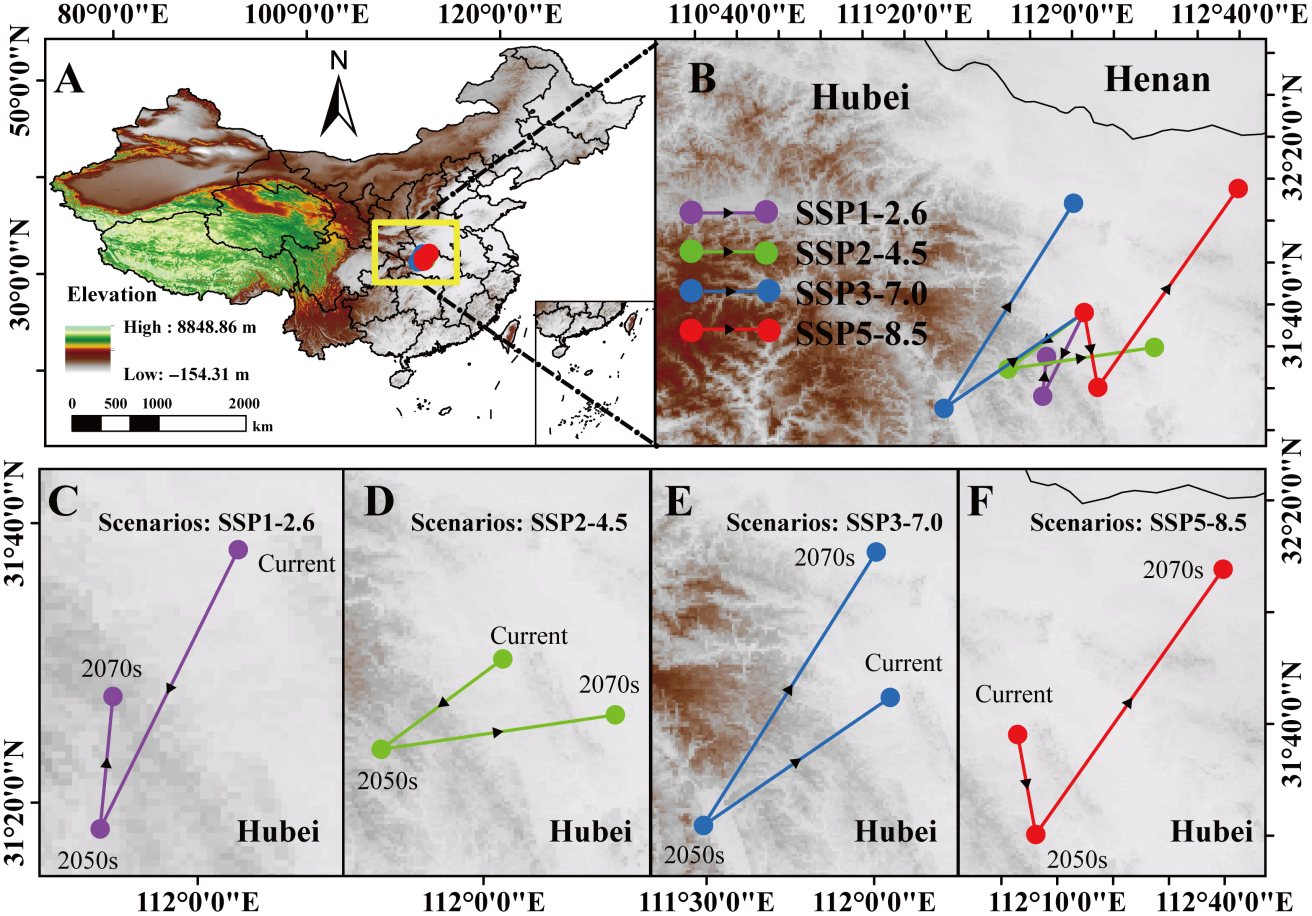
Transfer trajectories of potential distribution center routes for *Aromia bungii*. (A) Moving trajectories of potential distribution center routes for *A. bungii* in China. (B) The *A. bungii* movement routes under different shared socioeconomic path models. (C) SSP1‐2.6. (D) SSP2‐4.5. (E) SSP3‐7.0. (F) SSP5‐8.5.

The “Centroid Changes (Lines)” tool in ArcGIS Map software was utilized to compute alterations in the potential distribution center of total suitable habitat in the 2050s and 2070s, relative to the current potential distribution center, under four different future carbon emission scenarios (Figure 10). The objective is to identify general trends in distribution pattern changes for the future climate. The distribution center for *A. bungii* is currently located in Hubei Province at coordinates 31.634889° N, 112.048339° E (Figure 10B; Table S7).

In the SSP1‐2.6 pathway, the distribution center in the 2050s is located at 31.301910° N, 111.883438° E, and that in the 2070s is located at 31.253290° N, 111.490463° E, with the distribution center shifting 24.05 km to the southwest (Figure 10C; Table S5). In the SSP2‐4.5 pathway, the distribution center was located at 31.459798° N, 111.898860° E in the 2050s and 32.069108° N, 112.006272° E in the 2070s, shifting the center of distribution by 30.71 km to the southeast (Figure 10D; Table S7). The distribution center in the SSP3‐7.0 pathway was located at 31.410392° N, 111.746002° E in the 2050s and 31.337205° N, 112.102807° E in the 2070s and shifted to the north by 48.31 km (Figure 10E; Table S7). In the SSP5‐8.5 pathway, the distribution center for *A. bungii* was located at 31.496012° N, 112.328243° E in the 2050s and at 32.127851° N, 112.661427° E in the 2070s, with a northeastward shift of 79.70 km (Figure 10F; Table S7).

### 3.8 Dispersion of *A. bungii* Under Different Future Climate Scenarios

**FIGURE 11:**
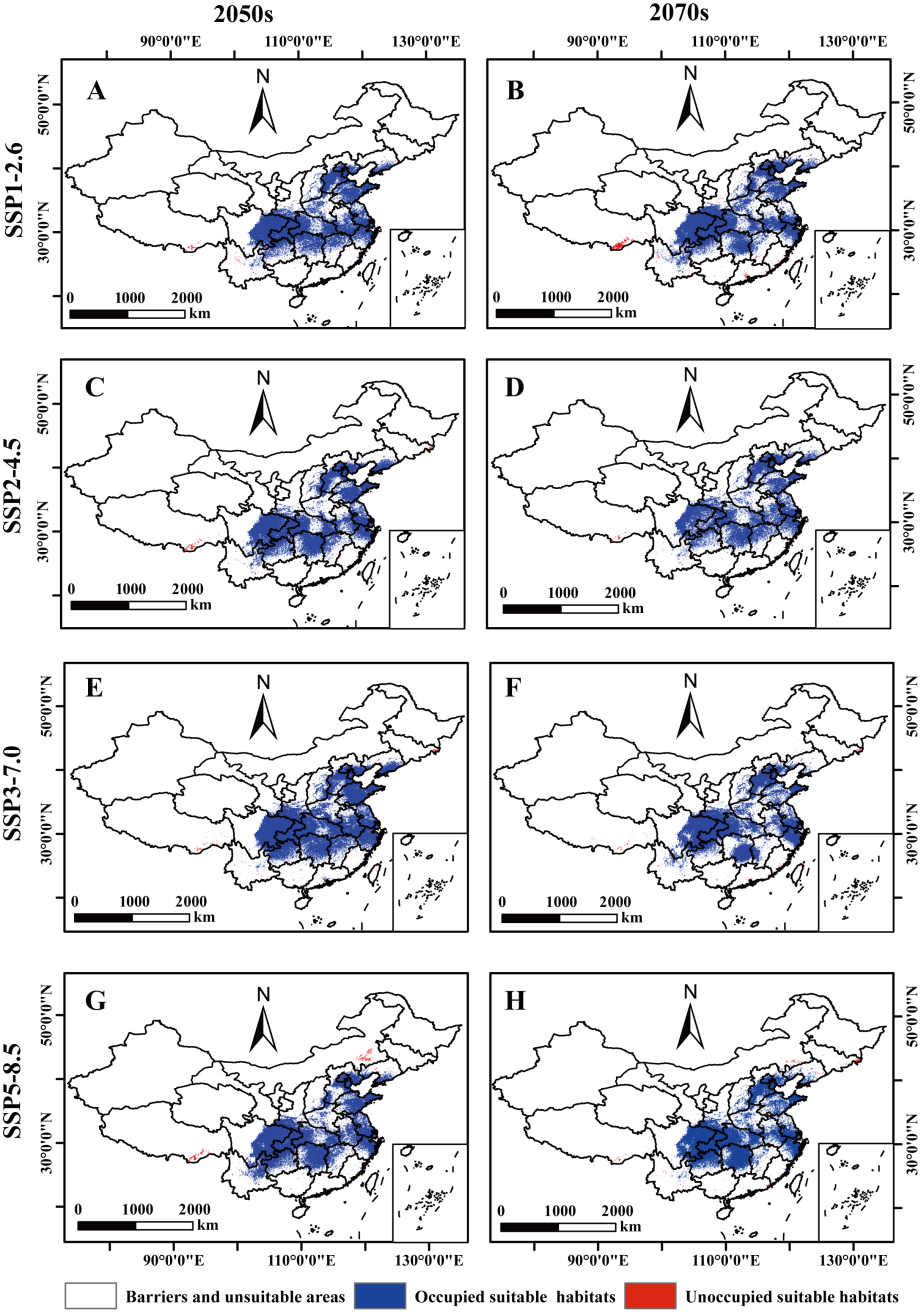
Occupied habitat maps of *Aromia bungii* under future climate scenarios. (A) SSP1‐2.5‐2050s; (B) SSP1‐2.5‐2070s; (C) SSP2‐4.5‐2050s; (D) SSP2‐4.5‐2070s; (E) SSP3‐7.0‐2050s; (F) SSP3‐7.0‐2070s; (G) SSP5‐8.5‐2050s; (H) SSP5‐8.5‐2070s.

The MigClim model projected the changes in the area and distribution of *A. bungii* under future climate scenarios. Barriers limiting its dispersal were mainly observed in the central and eastern provinces and cities, including Liaoning, Beijing, Tianjin, Hebei, Shanxi, Shandong, Henan, Shaanxi, Jiangsu, Shanghai, Anhui, Zhejiang, Hubei, Hunan, Chongqing, and Sichuan (Figure 11). Under the projected future climate scenarios, the area of unoccupied suitable habitat increased under all four climate scenarios, with the area of unoccupied suitable habitat for *A. bungii* ranging from 0.90 × 10^4^ km^2^ to 3.59 × 10^4^ km^2^, accounting for 0.09% to 0.37% of the total area (Table S8). The analysis of the area of suitable habitat revealed that under the three climate scenarios, the area of unoccupied suitable habitat decreased under the SSP2‐4.5, SSP3‐7.0, and SSP5‐8.5 pathways. However, the SSP1‐2.6 pathway showed an increasing trend in the area of unoccupied suitable habitat (Figure 11).

### 3.9 Changes in the Elevation of Suitable Areas for *A. bungii* Under Different Future Climate Scenarios

This study assumes that the elevation will remain constant in future years. Under the current climatic conditions, the suitable area elevation of *A. bungii* is 729.35 ± 2.27 m (Table 2). Under the SSP1‐2.6 pathway, the increase in the suitable area of *A. bungii* in the 2050s was 724.03 ± 2.071 m, which is a decrease of 5.32 m compared to the current suitable area elevation. However, the suitable area elevation in the 2070s was 792.31 ± 2.32 m, which is an increase of 62.96 m compared to the current suitable area elevation (Table 2). Under the SSP2‐4.5 pathway, the suitable area elevations of *A. bungii* in the 2050s and 2070s were 727.59 ± 2.10 and 706.86 ± 2.07 m, respectively, which are decreases of 1.77 and 22.49 m, respectively, compared to the current suitable area elevation (Table 2). In contrast to the SSP2‐4.5 pathway, under the SSP3‐7.0 pathway, the suitable area elevations of *A. bungii* in the 2050s and 2070s were 834.41 ± 2.37 and 747.98 ± 2.27 m, respectively, which represent increases of 105.06 and 18.63 m, respectively, compared to the current suitable area elevation (Table 2). Similarly, under the SSP5‐8.5 pathway, the suitable area elevation of *A. bungii* in the 2050s and 2070s was 727.58 ± 2.09 and 701.87 ± 2.13 m, respectively, a decrease of 1.0 m from the current suitable area elevation and a decrease of 1.77 and 27.49 m, respectively, from the current suitable area elevation (Table 2).

**TABLE 2:** Elevation distribution of *Aromia bungii* potential suitable areas under current and future climate scenarios.

Shared socioeconomic pathways	Elevation (m)	Elevation variation (m)
Current	729.35 ± 2.27	—
Future‐SSP1‐2.6 2040‐2060	724.03 ± 2.07	−5.32
Future‐SSP1‐2.6 2060‐2080	792.31 ± 2.32	62.96
Future‐SSP2‐4.5 2040‐2060	727.59 ± 2.10	−1.77
Future‐SSP2‐4.5 2060‐2080	706.86 ± 2.07	−22.49
Future‐SSP3‐7.0 2040‐2060	834.41 ± 2.37	105.06
Future‐SSP3‐7.0 2060‐2080	747.98 ± 2.27	18.63
Future‐SSP5‐8.5 2040‐2060	727.58 ± 2.09	−1.77
Future‐SSP5‐8.5 2060‐2080	701.87 ± 2.13	−27.49

## 4 Discussion

### 4.1 Analysis of Geospatial Distribution Patterns

This study analyzed the geospatial distribution pattern of *A. bungii* according to the first law of geography, which helps us have a comprehensive understanding of the spatial distribution of *A. bungii*. Moran's *I* provided the general distribution trend, while Getis‐Ord Gi* further revealed the local high‐risk areas. This information not only helps to understand the spatial dispersal pattern of *A. bungii*, but also provides a scientific basis for the development of more precise management and control strategies, especially for its possible dispersal trend in the context of future climate change, which can provide a basis for the government and the forestry sector to allocate resources and take measures more effectively. Moran's *I* index suggests that *A. bungii* exhibits spatial aggregation (100 × 100 km) within China. However, it was not possible to determine whether *A. bungii* was a high‐value or low‐value aggregation, and the Getis‐Ord General *G* index could compensate for the limitation of Moran's *I* index. The results showed that *A. bungii* in China was mainly a high‐value aggregation (Troiani et al. 2017; Ren, Shang, and Zhang 2020). Therefore, the results obtained by using both Moran's *I* index and the Getis‐Ord General *G* index show that there is a clear aggregation of *A. bungii* in China. Moreover, local Moran's *I* calculations revealed five areas in the high–high region, namely, Chongqing, Sichuan, Hubei, Jiangsu, and Hebei, and the areas surrounding the high distribution areas were also high distribution areas. Within these areas, the highly distributed areas are attracted to each other or are subject to common influences. Getis‐Ord Gi* indicates three hot spot distribution areas located in Hebei, Hubei, and Jiangsu. Analysis of the spatial distribution pattern of *A. bungii* revealed that the distribution points of *A. bungii* were mainly located in the Sichuan Basin, the Jianghan Plain, and the North China Plain. These areas have a flat and broad terrain and belong to the subtropical monsoon humid climate. Various varieties of forests and fruit trees have been planted in these areas, providing a suitable environment and important material basis for the survival of *A. bungii* (Wei et al. 2016; Shi et al. 2024). In addition, based on the results of Getis‐Ord Gi*, targeted prevention, control, and management strategies can be developed for different provinces and regions in China. Stricter monitoring and interventions can be prioritized for “hot spot” areas (Hebei, Hubei, and Jiangsu), while “cold spot” areas can be monitored and managed at a lower intensity. Although the distribution data in this study are richer than those in the literature and public online databases. However, the distribution data of *A. bungii* in this study may be incomplete, which is what led to the distribution points of *A. bungii* occurring mainly in the earlier areas. In the future, field surveys should continue to collect more comprehensive geographical information on the location. However, the spatial pattern of *A. bungii* analyzed in this study is still important for future species monitoring.

### 4.2 Analysis of the Main Bioclimatic Variables

Climatic factors are closely related to the individual development and population dynamics of insects, and temperature and precipitation are two important environmental factors that have a major impact on the potential distribution of insects (Bacci et al. 2021; Zhang et al. 2023). According to the MaxEnt model, the potential suitable habitat for *A. bungii* in China is mainly limited by human activities and climate variables, with the human factors (global human footprint and global human influence index), minimum temperature of coldest month (Bio6), and precipitation of wettest month (Bio13) being the most important variables affecting *A. bungii*. The larvae of *A. bungii* infest the branches of host plants. The ability to fly is lost by some insects of the family Cerambycidae when the ambient temperature is below 20°C (Chmura et al. 2011; Sutin et al. 2019). Therefore, temperature can directly affect their dispersal, geographical expansion, and geographical distribution within China (Schoettle et al. 2022). Furthermore, climate change is likely to cause a decrease in precipitation in the future, which will increase drought stress and reduce soil moisture (Green et al. 2019; Rafalska et al. 2023). This may prevent insects from reproducing, growing, and surviving. Human activities have a significant impact on the distribution of species, both by promoting and hindering changes in their habitats (Boyer and Rivault 2006). It is important to note that *A. bungii* grows mainly in forests and fruit trees, and the agricultural work that artisanal producers are mainly engaged in can directly affect the growth and reproduction of *A. bungii* (Yang et al. 2022a, 2022b). Moreover, human activities can also lead to changes in land use, for instance, the fragmentation of natural forest habitats due to factors such as road construction and urbanization (Xiong et al. 2023). This can result in a reduction in the area suitable for *A. bungii* survival. Additionally, the distribution of *A. bungii* may be closely linked to its natural enemies (predatory or parasitic), which hinder its rapid spread, and to current pest control efforts (Welch and Harwood 2014). Therefore, it can be inferred that human activities hinder the spread and propagation of *A. bungii*.

### 4.3 Geographical Distribution of Suitable Habitats for *A. bungii* Under Climate Change

Although *A. bungii* has a wide range of potential habitats, its distribution is mainly concentrated in the North China Plain, the middle and lower Yangtze River Plain, and the Sichuan Basin, including transverse mountain ranges such as the Taihang and Qinling Mountains. The Hengduan Mountains are sensitive to global climate change, with significant edge effects and high environmental heterogeneity (Feng et al. 2019). In the future, mountainous areas may provide opportunities for more species to survive, and *A. bungii*, a winged insect that can migrate, adapt to new environments, colonize and evolve in a relatively short period of time, and can adapt and spread in ecologically fragile mountainous areas (Cao et al. 2022; Chen et al. 2024). In addition, the geographic distribution pattern of *A. bungii* will change under future climate change scenarios, and the suitable area of *A. bungii* under any future carbon emission scenario will be larger than that in the current period. At the same time, the projected suitable area under the SSP1‐2.6 pathway will gradually increase over time, which confirms the adaptability of *A. bungii* to environmental changes. Understanding the movement of potential distribution patterns of *A. bungii* within China in the context of climate change is essential for developing conservation strategies to maintain ecological balance.

### 4.4 Migration of the Distribution Center of *A. bungii*

Climate change not only affects the extent of a species' potential range, but also causes the center of its range to shift (Xu et al. 2024). When changes in these climatic factors reach or exceed the suitable intervals required for the growth of species, the geographical distribution of the species will shift (Low et al. 2021; Campos et al. 2023). The results of the analysis of the center of the distribution range showed that *A. bungii* migrated to higher latitudes in the SSP3‐7.0 and SSP5‐8.5 scenarios. However, species migrated to lower latitudes under the SSP1‐2.6 and SSP2‐4.5 scenarios. Overall, migration direction and migration distance varied across scenarios, possibly due to varying degrees of future warming (Poggio, Simonetti, and Gimona 2018). Second, the movement to lower latitudes is relative and may simply be due to the degradation of some of the suitable zones, resulting in movement in favor of lower latitudes. In most cases, the trend toward lower latitudes is more due to geographical anomalies than to physiological aspects of the species under climate change (Sun et al. 2020). Alternatively, *A. bungii* may also be related to broader requirements for elevation and temperature conditions. Currently, human‐mediated migration is common for many species due to the increasing impact of human activities on ecosystems, which may help them overcome the problem of migration distance. Our findings are of practical and theoretical significance for forest conservation and prevention of *A. bungii* hazards and are important for understanding responses to future climate change and developing coping strategies.

### 4.5 Dispersion of *A. bungii* Under Climate Change

Forest pests are expected to occupy larger habitats under future climate scenarios (Wang et al. 2019). The area of unoccupied suitable habitat for *A. bungii* gradually increased with increasing CO_2_ concentration in a time‐dependent manner. According to the SSP1‐2.6 pathway, CO_2_ emissions will continue to decrease in the future, resulting in a positive growth of the habitat occupied by *A. bungii* over time. However, under the medium‐high carbon emission scenarios (SSP2‐4.5, SSP3‐7.0, and SSP5‐8.5), the occupied habitat area gradually decreased with time but still showed positive growth. Furthermore, due to climate change, forested areas in northeastern and southwestern China (Qinghai–Tibet Plateau, Taihang Mountains, and Nanling Mountains, etc.) will become more suitable for the growth of *A. bungii*, and the potential habitat of *A. bungii* will gradually shift to higher altitudes. Climate change is predicted to impact the distribution of occupied *A. bungii* habitats in the future, with varying effects by region.

### 4.6 Recommendations for the Prevention and Control of Pests

Spatial geographic tools and modeling methods can be used to determine the distribution areas of forest pests, and this information can help government managers and decision makers formulate rational forest management plans (Arabameri, Pourghasemi, and Yamani 2017; Mishra et al. 2024). These recommendations are valuable for maintaining the health and safety of forest ecosystems and controlling pests. Climate change will increase the probability of *A. bungii* suitability, which poses a threat to forest ecological security and sustainable development of the fruit industry for human health within the forest area (Marchioro and Krechemer 2021). Therefore, forest managers are advised to take additional precautionary measures in areas with high pest suitability. A combination of chemical and physical control methods should be used, along with the planting of insect‐resistant forest saplings and the rationalization of pest control in areas with medium to low suitability. In nonsuitable areas, no control should be carried out to conserve biodiversity. Simultaneously, it is essential to log trees reasonably and strictly control logging areas (Hyvärinen et al. 2005). Moreover, it may negatively affect the stability of the forest community structure, cause fragmentation of natural habitats, and exacerbate the spread of pests (Pierri‐Daunt and Tanaka 2014; Ostalé‐Valriberas et al. 2018). Additionally, it is crucial to enhance the promotion and popularization of science and technology in forest areas and implement forest ecological protection policies scientifically and effectively (Railsback and Johnson 2014).

### 4.7 Limitations of This Research

Future climate change is associated with many uncertainties. This study only uses the BCC‐CMS2‐MR climate model to simulate the potential suitable areas of *A. bungii* within China. Previous studies have employed multiple global climate models, and future studies on predicting potential suitable habitat for *A. bungii* should also use multiple global climate models (Nguyen et al. 2022). This will provide decision makers with a wider range of options for developing forest ecological conservation measures in practice. It is important to note that this study only considered the effects of abiotic factors (31 environmental variables) on species distribution (Santana et al. 2019; Maruthadurai, Das, and Ramesh 2023). In reality, biotic factors such as competition, predation, and disease also affect species distributions (Rostro‐García et al. 2016; Fern et al. 2019). To consider these factors, it will be necessary to build a more complex and integrated SDMs for simulation. This will indicate the most important direction for future model development.

## 5 Conclusions

In this article, a comprehensive SDM (including geospatial, MaxEnt, and MigClim) was developed, taking into account environmental variables for climate, topography, UV radiation, and human activities, to explore the extent of the spatial geographic distribution of *A. bungii* in China, and to provide a basis for evaluating its potentially suitable and unoccupied suitable habitats. The results of the study showed that the AUC values of the MaxEnt model were all higher than 0.9, indicating that the optimized MaxEnt model has excellent performance and a high level of accuracy. Human activities will inhibit the expansion of suitable habitats for *A. bungii* in China, however, future climate change will facilitate the expansion of *A. bungii*, expansion occurred primarily in the central and southwestern regions of China. Moreover, unoccupied suitable habitat for *A. bungii* will continue to increase in the Tibetan Plateau and Taihang Mountains regions of China. These findings have practical implications for safeguarding forest health and promoting ecological sustainability in China, as well as for implementing effective control measures under changing climatic conditions.

## Author Contributions

**Liang Zhang:** conceptualization (equal), methodology (equal), software (equal), writing – original draft (equal), writing – review and editing (equal). **Ping Wang:** investigation (equal), supervision (equal), writing – review and editing (equal). **Guanglin Xie:** investigation (equal), supervision (equal), writing – review and editing (equal). **Wenkai Wang:** funding acquisition (lead), supervision (lead), writing – review and editing (lead).

## Conflicts of Interest

The authors declare no conflicts of interest.

## Supporting information


Data S1.

## Data Availability

The original contributions presented in the study are included in the article/Supporting Information. Further inquiries can be directed to the corresponding authors.
