# Supplementary material for: Spatial Distribution Pattern of Aromia bungii Within China and Its Potential Distribution Under Climate Change and Human Activity
Source: Ecol Evol. 2024 Nov 13;14(11):e70520. doi: 10.1002/ece3.70520 (PMC11560860; doi:10.1002/ece3.70520)
Supplement: Supplementary file 1 — Data S1. [file ECE3-14-e70520-s001.zip › Supplementary Materials.docx]

**Spatial distribution pattern of *Aromia bungii* within China and its potential distribution under climate change and human activity**

**Supplementary data**

**
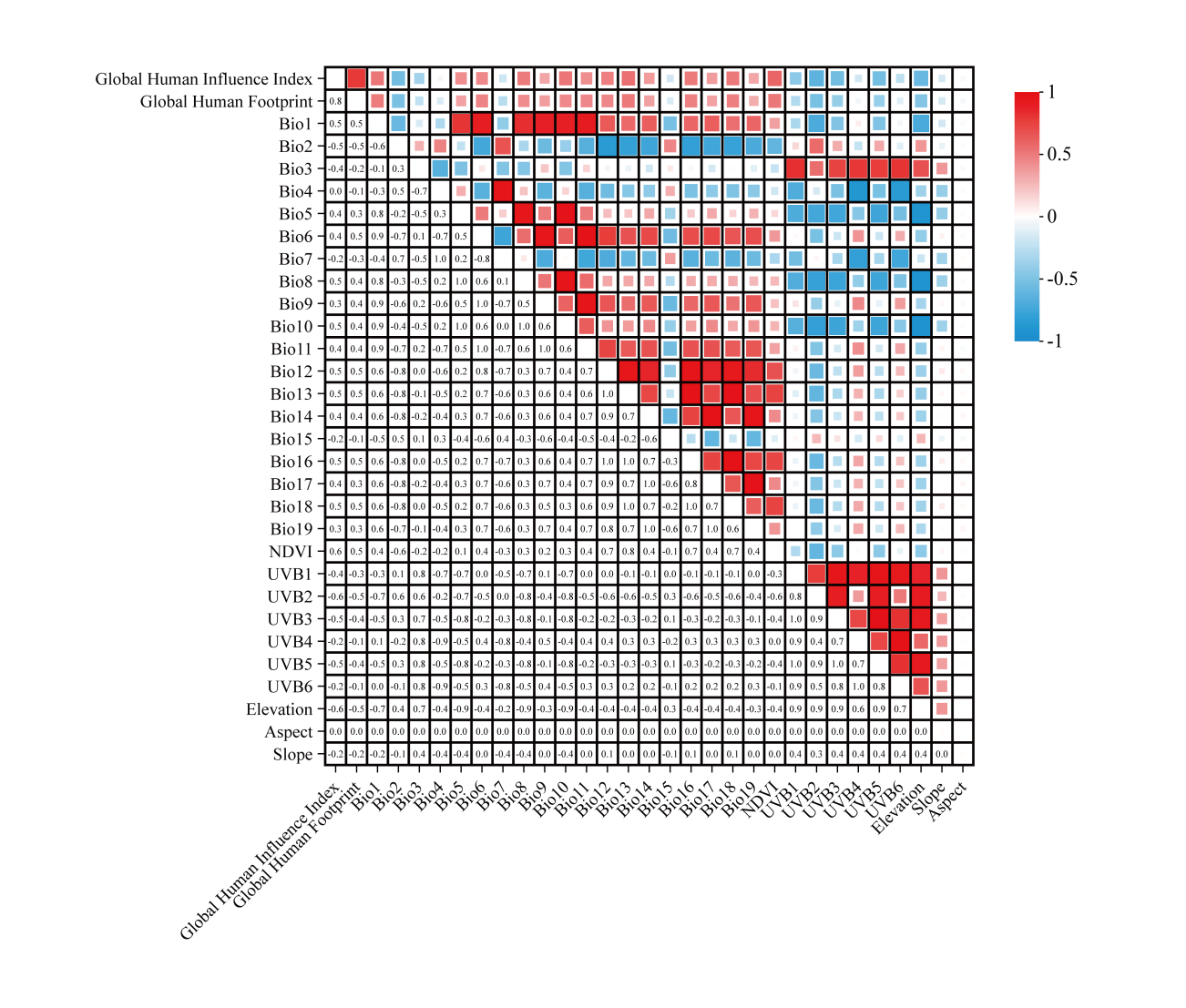
**

**Figure S1.** Correlation among the 31 bioclimatic variables.


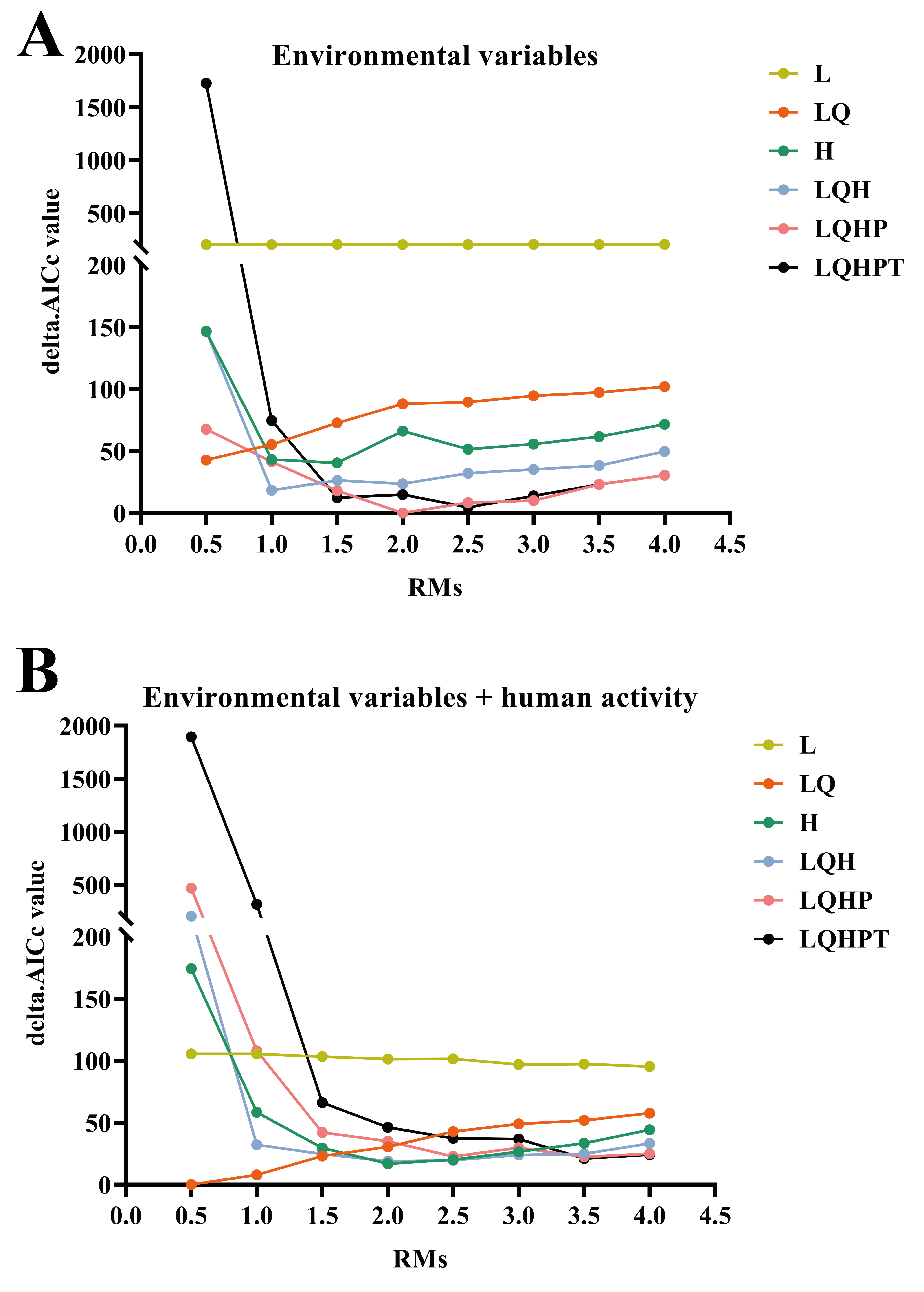


**Figure S2.** Tuning parameters for *Aromia bungii* in predicting potential distribution regions using the MaxEnt model. (A) Environmental variables. (B) Environmental variables + human activity.

**TABLE S1**

Correlation analysis and screening of environmental variables.

| Abbreviation | Environmental variables | Operation (\|*r*\| > 0.9) |
| --- | --- | --- |
| Bio1 | Annual mean temperature (◦C) | Eliminate |
| Bio2 | Mean diurnal range (◦C) | Retain |
| Bio3 | Isothermality | Retain |
| Bio4 | Temperature seasonality | Retain |
| Bio5 | Maximum temp of warmest month (◦C) | Eliminate |
| Bio6 | Minimum temp of coldest month (◦C) | Retain |
| Bio7 | Temperature annual range (◦C) | Eliminate |
| Bio8 | Mean temp of wettest quarter (◦C) | Eliminate |
| Bio9 | Mean temp of driest quarter (◦C) | Eliminate |
| Bio10 | Mean temp of warmest quarter (◦C) | Eliminate |
| Bio11 | Mean temp of coldest quarter (◦C) | Eliminate |
| Bio12 | Annual precipitation (mm) | Eliminate |
| Bio13 | Precipitation of wettest month (mm) | Retain |
| Bio14 | Precipitation of driest month (mm) | Retain |
| Bio15 | Precipitation seasonality (mm) | Retain |
| Bio16 | Precipitation of wettest quarter (mm) | Eliminate |
| Bio17 | Precipitation of driest quarter (mm) | Eliminate |
| Bio18 | Precipitation of warmest quarter (mm) | Eliminate |
| Bio19 | Precipitation of coldest quarter (mm) | Eliminate |
| Bio20 | Elevation (m) | Eliminate |
| Bio21 | NDVI | Retain |
| Bio22 | Slope | Retain |
| Bio23 | Aspect | Retain |
| Bio24 | Annual_mean_UV-B | Eliminate |
| Bio25 | UV-B_seasonality | Eliminate |
| Bio26 | Mean_UV-B_of_highest_month | Eliminate |
| Bio27 | Mean_UV-B_of_lowest_month | Retain |
| Bio28 | Sum_of_UV-B_radiation_of_highest_quarter | Eliminate |
| Bio29 | Sum_of_UV-B_radiation_of_lowest_quarter | Eliminate |
| Bio30 | Global human footprint | Retain |
| Bio31 | Global human influence index | Retain |

**TABLE S2**

The global Moran’s I report for *Aromia bungii*.

| Global Moran’s I Index | Expectation Index | Variance | z-score | *p* |
| --- | --- | --- | --- | --- |
| 0.161080 | -0.000592 | 0.000139 | 13.715704 | < 0.05 |

**TABLE S3**

# The Getis-Ord General G report for *A. bungii*.

| Getis-Ord General G Index | Expectation Index | Variance | z-score | *p* |
| --- | --- | --- | --- | --- |
| 0.044963 | 0.004285 | 0.000009 | 13.862348 | < 0.05 |

**TABLE S4**

Area of suitable habitat for *A. bungii* under current (with and without human activity disturbance) and future climate scenarios.

| Shared socioeconomic pathways | Predicted area (10^4^ km^2^) | | | Comparison with Current-Environmental variables distribution (%) | | |
| --- | --- | --- | --- | --- | --- | --- |
|  | Low suitable | Medium suitable | High suitable | Low suitable | Medium suitable | High suitable |
| Current-Environmental variables | 118.08 | 93.73 | 34.61 | - | - | - |
| Current-Environmental variables + Human activity | 138.40 | 46.95 | 18.32 | 17.21 | -49.90 | -47.08 |
| Future-SSP1-2.6 2040-2060 | 124.77 | 98.85 | 30.39 | 5.67 | 5.46 | -12.19 |
| Future-SSP1-2.6 2060-2080 | 134.96 | 95.32 | 36.65 | 14.30 | 1.70 | 5.90 |
| Future-SSP2-4.5 2040-2060 | 132.16 | 94.97 | 33.79 | 11.92 | 1.32 | -2.35 |
| Future-SSP2-4.5 2060-2080 | 129.98 | 92.76 | 27.72 | 10.08 | -1.04 | -19.92 |
| Future-SSP3-7.0 2040-2060 | 131.36 | 114.50 | 43.78 | 11.25 | 22.16 | 26.51 |
| Future-SSP3-7.0 2060-2080 | 134.94 | 86.15 | 35.20 | 14.28 | -8.09 | 1.70 |
| Future-SSP5-8.5 2040-2060 | 125.24 | 104.60 | 34.07 | 6.07 | 11.60 | -1.55 |
| Future-SSP5-8.5 2060-2080 | 125.47 | 92.81 | 34.63 | 6.26 | -0.98 | 0.08 |

**TABLE S5**

Relative change in potential range size of *A. bungii* under future climate scenarios.

| Shared socioeconomic pathways | Predicted area (10^4^ km^2^) | | | |
| --- | --- | --- | --- | --- |
|  | Expansion | No occupancy | Unchanged | Contraction |
| SSP1-2.6 2040-2060 | 24.57 | 680.55 | 229.44 | 14.39 |
| SSP1-2.6 2060-2080 | 29.82 | 675.31 | 237.12 | 6.712 |
| SSP2-4.5 2040-2060 | 28.44 | 676.68 | 232.48 | 11.36 |
| SSP2-4.5 2060-2080 | 18.16 | 686.96 | 232.30 | 11.53 |
| SSP3-7.0 2040-2060 | 48.70 | 656.43 | 240.95 | 2.88 |
| SSP3-7.0 2060-2080 | 24.09 | 681.04 | 232.20 | 11.63 |
| SSP5-8.5 2040-2060 | 28.76 | 676.36 | 235.15 | 8.68 |
| SSP5-8.5 2060-2080 | 18.60 | 686.53 | 234.32 | 9.51 |

**TABLE S6**

Multivariate environmental similarity surfaces (MESS) affecting the distribution of *A. bungii* under future climate conditions.

| Shared socioeconomic pathways | S value area (10^4^ km^2^) | | | | |
| --- | --- | --- | --- | --- | --- |
|  | S ≤ 0 | 0 < S ≤ 10 | 10 < S ≤ 20 | 20 < S ≤ 30 | S ≥ 30 |
| SSP1-2.6 2040-2060 | 4.94 | 590.09 | 191.81 | 102.43 | 59.68 |
| SSP1-2.6 2060-2080 | 5.61 | 629.32 | 164.27 | 88.46 | 61.30 |
| SSP2-4.5 2040-2060 | 3.48 | 606.55 | 182.38 | 95.28 | 61.27 |
| SSP2-4.5 2060-2080 | 2.92 | 624.06 | 167.78 | 90.15 | 64.04 |
| SSP3-7.0 2040-2060 | 1.28 | 614.40 | 172.23 | 96.43 | 64.61 |
| SSP3-7.0 2060-2080 | 3.16 | 627.24 | 156.02 | 91.55 | 70.98 |
| SSP5-8.5 2040-2060 | 3.07 | 646.60 | 157.33 | 82.04 | 59.92 |
| SSP5-8.5 2060-2080 | 10.84 | 651.69 | 146.49 | 81.88 | 58.06 |

**TABLE S7**

Alternational trends of longitude, latitude and migration distance of *A. bungii* in different periods.

| Shared socioeconomic pathways | Longitude (°E) | Latitude (°N) | Center migration distance (km) |
| --- | --- | --- | --- |
| Current | 112.048339 | 31.634889 | - |
| Future-SSP1-2.6 2040-2060 | 111.883438 | 31.301910 | 40.11 |
| Future-SSP1-2.6 2060-2080 | 111.490463 | 31.253290 | 24.05 |
| Future-SSP2-4.5 2040-2060 | 111.898860 | 31.459798 | 38.00 |
| Future-SSP2-4.5 2060-2080 | 112.006272 | 32.069108 | 30.71 |
| Future-SSP3-7.0 2040-2060 | 111.746002 | 31.410392 | 67.84 |
| Future-SSP3-7.0 2060-2080 | 112.102807 | 31.337205 | 48.31 |
| Future-SSP5-8.5 2040-2060 | 112.328243 | 31.496012 | 33.41 |
| Future-SSP5-8.5 2060-2080 | 112.661427 | 32.127851 | 79.70 |

**TABLE S8**

Potential distribution area in different periods under the condition of barrier diffusion.

| Shared socioeconomic pathways | Predicted area (10^4^ km^2^) | | | Comparison with current distribution (%) | | | |
| --- | --- | --- | --- | --- | --- | --- | --- |
|  | Barriers and unsuitable areas | Occupied suitable habitats | Unoccupied suitable habitat | Barriers and unsuitable areas | Occupied suitable habitats | | Unoccupied suitable habitat |
| SSP1-2.6 2040-2060 | 832.18 | 128.20 | 1.40 | 86.53 | 13.33 | 0.15 | |
| SSP1-2.6 2060-2080 | 829.45 | 128.74 | 3.59 | 86.24 | 13.39 | 0.37 | |
| SSP2-4.5 2040-2060 | 832.69 | 127.42 | 1.66 | 86.58 | 13.25 | 0.17 | |
| SSP2-4.5 2060-2080 | 840.90 | 119.97 | 0.90 | 87.43 | 12.47 | 0.09 | |
| SSP3-7.0 2040-2060 | 803.14 | 156.55 | 2.08 | 83.51 | 16.28 | 0.22 | |
| SSP3-7.0 2060-2080 | 840.16 | 120.09 | 1.52 | 87.36 | 12.49 | 0.16 | |
| SSP5-8.5 2040-2060 | 822.72 | 136.55 | 2.50 | 85.54 | 14.20 | 0.26 | |
| SSP5-8.5 2060-2080 | 834.03 | 125.72 | 2.02 | 86.72 | 13.07 | 0.21 | |
